# Alkaloids from the Mangrove-Derived Actinomycete *Jishengella endophytica* 161111

**DOI:** 10.3390/md12010477

**Published:** 2014-01-21

**Authors:** Pei Wang, Fandong Kong, Jingjing Wei, Yi Wang, Wei Wang, Kui Hong, Weiming Zhu

**Affiliations:** 1 Key Laboratory of Marine Drugs, Ministry of Education of China, School of Medicine and Pharmacy, Ocean University of China, Qingdao 266003, China; E-Mails: wangpei850212@163.com (P.W.); kongfandong501@126.com (F.K.); wangyi0213@ouc.edu.cn (Y.W.); wwwakin@ouc.edu.cn (W.W.); 2 Key Laboratory of Combinatorial Biosynthesis and Drug Discovery, Ministry of Education, School of Pharmaceutical Sciences, Wuhan University, Wuhan 430071, China; E-Mail: rxwzq@163.com

**Keywords:** mangrove, actinomycete, *Jishengella endophytica* 161111, pyrazine derivative, anti-H1N1 virus activity

## Abstract

A new alkaloid, 2-(furan-2-yl)-6-(2*S*,3*S*,4-trihydroxybutyl)pyrazine (**1**), along with 12 known compounds, 2-(furan-2-yl)-5-(2*S*,3*S*,4-trihydroxybutyl)pyrazine (**2**), (*S*)-4-isobutyl-3-oxo-3,4-dihydro-1*H*-pyrrolo[2,1-*c*][1,4]oxazine-6-carbaldehyde (**3**), (*S*)-4-isopropyl-3-oxo-3,4-dihydro-1*H*-pyrrolo[2,1-*c*][1,4]oxazine-6-carbaldehyde (**4**), (4*S*)-4-(2-methylbutyl)-3-oxo-3,4-dihydro-1*H*-pyrrolo[2,1-*c*][1,4]oxazine-6-carbaldehyde (**5**), (*S*)-4-benzyl-3-oxo-3,4-dihydro-1*H*-pyrrolo[2,1-*c*][1,4]oxazine-6-carbaldehyde (**6**), flazin (**7**), perlolyrine (**8**), 1-hydroxy-β-carboline (**9**), lumichrome (**10**), 1*H*-indole-3-carboxaldehyde (**11**), 2-hydroxy-1-(1*H*-indol-3-yl)ethanone (**12**), and 5-(methoxymethyl)-1*H*-pyrrole-2-carbaldehyde (**13**), were isolated and identified from the fermentation broth of an endophytic actinomycetes, *Jishengella endophytica* 161111. The new structure **1** and the absolute configurations of **2**–**6** were determined by spectroscopic methods, *J*-based configuration analysis (JBCA) method, lactone sector rule, and electronic circular dichroism (ECD) calculations. Compounds **8**–**11** were active against the influenza A virus subtype H1N1 with *IC_50_* and selectivity index (*SI*) values of 38.3(±1.2)/25.0(±3.6)/39.7(±5.6)/45.9(±2.1) μg/mL and 3.0/16.1/3.1/11.4, respectively. The *IC_50_* and *SI* values of positive control, ribavirin, were 23.1(±1.7) μg/mL and 32.2, respectively. The results showed that compound **9** could be a promising new hit for anti-H1N1 drugs. The absolute configurations of **2**–**5**, ^13^C nuclear magnetic resonance (NMR) data and the specific rotations of **3**–**6** were also reported here for the first time.

## 1. Introduction

**Figure 1:**
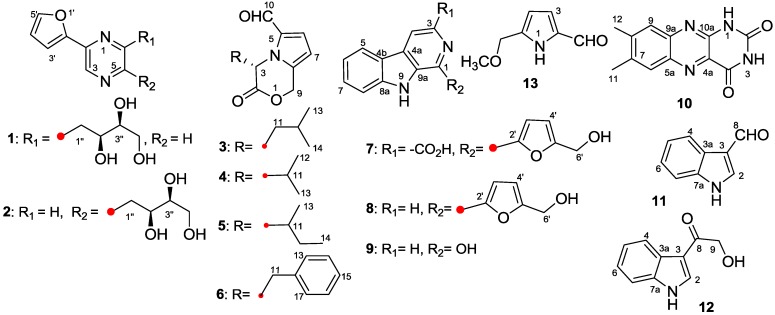
Chemical structures of compounds **1**–**13** from *J. endophytica* 161111.

Mangroves, unique forest ecosystems found mainly in the tropical and subtropical intertidal regions, represent a rich biological diversity and a high population of actinomycetes [1,2]. From these actinomycetes, many bioactive compounds such as cytotoxic streptocarbazoles A and B have been obtained [3]. In addition, there is also evidence that the mangrove ecosystem is a largely unexplored source for novel actinomycetes with the potential to produce biologically active secondary metabolites [4]. In order to pursue bioactive products from mangrove actinomycetes, a novel endophytic actinomycetes, identified as *Jishengella endophytica* 161111, had been isolated from the root of the mangrove plant, *Xylocarpus granatum* (Meliaceae) [5]. *J. endophytica* 161111 was found to produce alkaloids in a saline culture medium by thin layer chromatography (TLC) visualizing with Dragendorff’s reagent. Chemical investigations on the ethyl acetate (EtOAc) extract of fermentation broth of strain 161111 resulted in the isolation and identification of three pyrazine derivatives, 2-(furan-2-yl)-6-(2*S*,3*S*,4-trihydroxybutyl)pyrazine (**1**) and 2-(furan-2-yl)-5-(2*S*,3*S*,4-trihydroxybutyl) pyrazine (**2**) [6] and lumichrome (**10**) [7]; four pyrrololactones, (*S*)-4-isobutyl-3-oxo-3,4-dihydro-1*H*-pyrrolo[2,1-*c*][1,4]oxazine-6-carbaldehyde (**3**) [8], (*S*)-4-isopropyl-3-oxo-3,4-dihydro-1*H*-pyrrolo[2,1-*c*][1,4]oxazine-6-carbaldehyde (**4**) [8], (4*S*)-4-(2-methylbutyl)-3-oxo-3,4-dihydro-1*H*-pyrrolo[2,1-*c*][1,4]oxazine-6-carbaldehyde (**5**) [8,9] and (*S*)-4-benzyl-3-oxo-3,4-dihydro-1*H*-pyrrolo[2,1-*c*][1,4]oxazine-6-carbaldehyde (**6**) [10]; three β-carbolines, flazin (**7**) [11], perlolyrine (**8**) [12] and 1-hydroxy-β-carboline (**9**) [13]; along with 1*H*-indole-3-carboxaldehyde (**11**) [14,15], 2-hydroxy-1-(1*H*-indol-3-yl) ethanone (**12**) [14,15], and 5-(methoxymethyl)-1*H*-pyrrole-2-carbaldehyde (**13**) [16] (Figure 1). Compounds **8**–**11** showed anti-influenza A (H1N1) virus activity with the half maximal inhibitory concentration (*IC_50_*) and selectivity index (*SI*) values of 38.3(±1.2)/25.0(±3.6)/39.7(±5.6)/45.9(±2.1) μg/mL and 3.0/16.1/3.1/11.4, respectively. Pyrazine alkaloids from marine organisms exhibited cytotoxic and antimicrobial activities [17,18,19,20,21], three of which were identified from the mangrove plant [22] and fungi [23,24]. β-Carbolines, as a type of natural indol alkaloids, displayed cytotoxic [25], antiviral [26,27], antimicrobial [28], antiparasitic [29], and antithrombotic activities [30]. Apart from the terrestrial organisms, marine organisms, including mangrove fungi [24,31], are also a major source of β-carbolines [32,33,34,35,36,37,38,39,40]. Pyrrololactones that had been reported as the volatile components of the roasted roots of *Cichorium intrybus* [8] were not identified from the marine organisms.

## 2. Results and Discussion

### 2.1. Structure Elucidation

The EtOAc extract of the fermentation broth of *J. endophytica* 161111 was subjected to extensive chromatographic separations over silica gel, RP-18, Sephadex LH-20 and high performance liquid chromotography (HPLC) to yield the new compound **1** and the known compounds **2**–**13**.

**Figure 2:**
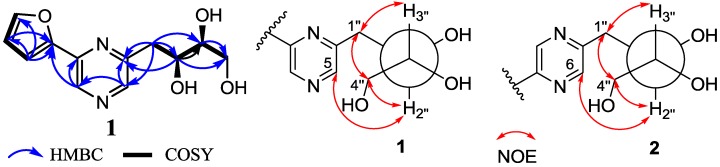
Selected 2D NMR correlations for **1** and Newman projections showing NOESY correlations and ^3^ *J*_H-2″,H-3″_ values of **1** and **2**.

**Figure 3:**
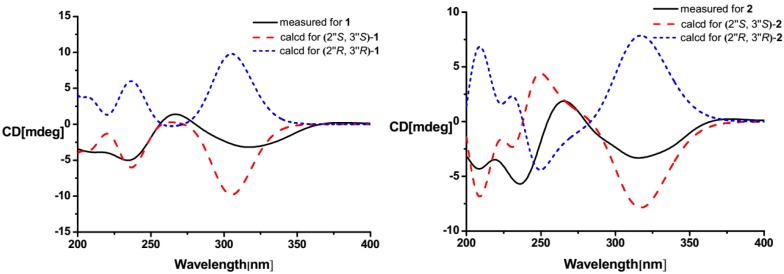
CD and calculated ECD spectra for **1** and **2**.

Compound **1** was obtained as brown oil. Its molecular formula was determined as C_12_H_14_N_2_O_4_ on the basis of high resolution electrospray ionization mass spectrum (HRESIMS) peak at *m*/*z* 273.0849 [M + Na]^+^ (calcd. for C_12_H_14_N_2_O_4_Na 273.0846). The similarity of 1D- and 2D-NMR data (Table S2 in Supplementary Information) between **1** and the known crotonine (**2**) [6] indicated the similar planar structure. The signals at δ_H_ 7.23 (1H, d, *J* = 4.0 Hz), 6.63 (1H, dd, *J* = 2.1, 4.0 Hz) and 7.72 (1H, d, *J* = 2.1 Hz) revealed the presence of a α-substituted furan moiety, which was further identified by the ^1^H-^1^H correlation spectroscopy (COSY) of H-3′/H-4′/H-5′ and the key heteronuclear multiple bond correlations (HMBC) of H-3′ (δ 7.23) to C-2′ (δ 151.1), C-4′ (δ 112.0) and C-5′ (δ 144.6), of H-4′ (δ_H_ 6.63) to C-2′, C-3′ (δ 110.5) and C-5′, and of H-5′ (δ 7.72) to C-2′ and C-3′. The existence of 2,3,4-trihydroxybutyl moiety was deduced from the ^1^H-^1^H COSY correlations of H-1″/H-2″/H-3″/H-4″ along with the key HMBC correlations of H-1″ (δ 3.24/2.93) to C-2″ (δ 71.5) and C-3″ (δ 74.8), H-2″ (δ 4.03) to C-3″ and C-4″ (δ 63.2), H-3″ (δ 3.58) to C-1″ (δ 38.4), C-2″ and C-4″, and H-4″ (δ 3.80/3.65) to C-2″ and C-3″. The remainder C_4_H_2_N_2_ displayed two sp^2^ methine signals at δ_H/C_ 8.78/136.7 and 8.38/142.7, and two sp^2^ quaternary carbon signals at δ_C_ 144.2 and 155.3, which were ascribable to a disubstituted pyrazine nucleus [41]. The obvious HMBC correlations from H-3 (δ 8.78) to C-2 (δ 144.2) and C-2′, H-5 (δ 8.38) to C-3 (δ 136.7) and C-6 (δ 155.3), and from H-2″ to C-5 (δ 142.7) and C-6 indicated that the α-substituted furan moiety and the 2,3,4-trihydroxybutyl moiety were linked onto C-2 and C-6 of the pyrazine nucleus, respectively. The relative configuration of **1** was determined by *J*-based configuration analysis (JBCA) method [42]. The low temperature NMR (−4 °C) of **1** revealed a large coupling constant between H-2″ and H-3″ (*J* = 7.2 Hz), indicating an *anti*-relationship between the two protons. In addition, low temperature NMR (−4 °C) of **1** also displayed nuclear Overhauser effect spectroscopy (NOESY) of H-1″/H-3″, H-1″/H-4″, and H-4″/H-2″. These data indicated *threo*-configuration between C-2″ and C-3″ (Figure 2). The absolute configuration of **1** was determined by use of quantum chemical ECD calculation [43]. The preliminary conformational distribution search was performed by HyperChem 7.5 software. The corresponding minimum geometries were further fully optimized by using density functional theory (DFT) at the B3LYP/6-31G(d) level as implemented in the Gaussian 03 program package. The stable conformers obtained were submitted to ECD calculation by the time-dependent DFT (TDDFT) (B3LYP/6-31G(d)) method. The overall predicted ECD spectrum of **1** was subsequently compared with the measured one [43]. The measured circular dichroism (CD) curve of **1** showed Cotton effect at λ_max_ (Δε) 310 (−0.14), 284 (+0.12) and 237 (−1.3) nm, matching well with the calculated ECD curve of (2″*S*,3″*S*)-**1** (Figure 3). Thus, the new structure, **1**, was established as 2-(furan-2-yl)-6-(2*S*,3*S*,4-trihydroxybutyl)pyrazine.

Although the planar structures of **2**–**5** had reported in the literatures [6,8,9], their absolute configurations have not been resolved yet. The planar structures of **2** and **6** here were elucidated by comparison of their NMR data with those reported [6,10]. Then, the same methods as **1** were used to resolve the relative and absolute configurations of **2**. The large ^3^ *J*_H-2″,H-3″_ value (7.1 Hz) and the NOESY correlations of H-1″/H-3″ and H-1″/H-4″/H-2″ in the low temperature NMR (−4 °C) of **2** revealed the same *threo*-configuration of two hydroxy groups at C-2″ and C-3″ (Figure 2). Compound **2** showed a CD Cotton effect at λ_max_ (Δε) 316 (−0.54), 267 (+0.35) and 235 (−1.0) nm, matching with the calculated ECD curve of (2″*S*,3″*S*)-**2** (Figure 3). Thus, compound **2** was determined as 2-(furan-2-yl)-5-(2*S*,3*S*,4-trihydroxybutyl)pyrazine.

**Figure 4:**
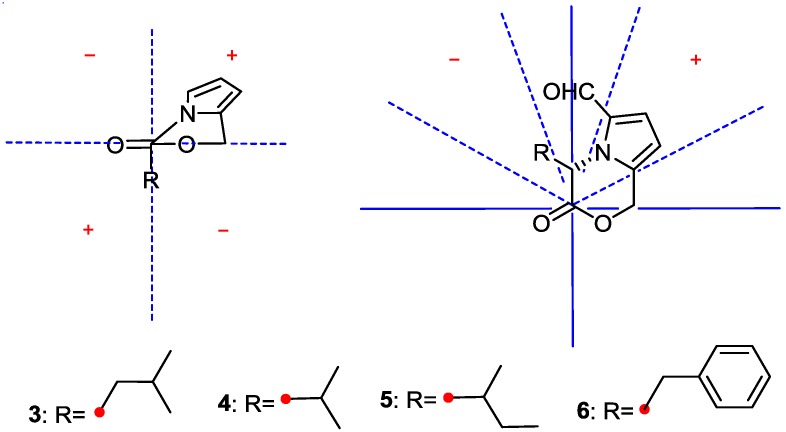
Application of lactone sector rule to **3**–**6**.

**Figure 5:**
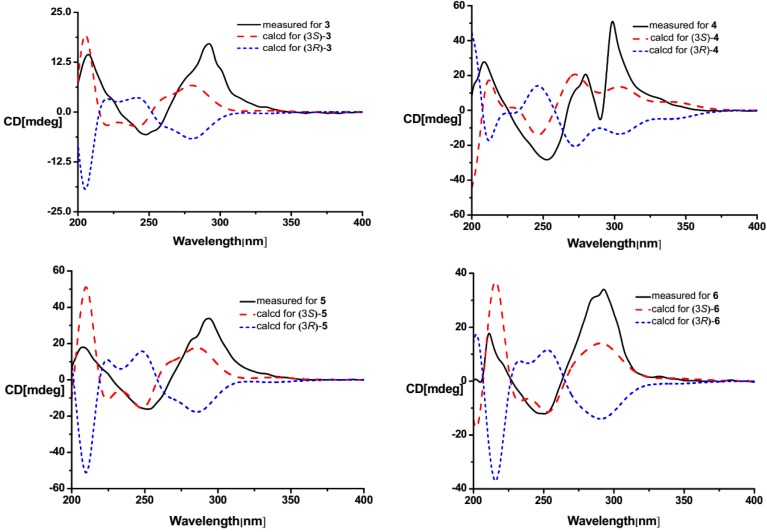
CD and calculated ECD spectra of **3**–**6**.

Though absolute structure **6** had been presented in the literature [10], no reference data on absolute configuration, such as CD and specific rotation of **6**, could be used. Lactone sector rule [44] could be used to determine the absolute configuration of **6**. The molecule was viewed from the line on the plane of the ester group along the bisectrix of the O–C=O angle, *i.e.*, the line from C-2 to C-3 as shown in Figure 4 for (3*S*). The functional group at C-3 lying in the back upper left sector was responsible for the positive CD Cotton effect resulted from n–π* transition of lactone, which was well in accordance with the positive Cotton effect at λ_max_ 293 nm of **6** (Figure 4). The deduction was further confirmed by ECD quantum chemical calculations using the TDDFT method at the B3LYP/6-31G(d) level in Gaussian 03 [43]. The measured CD curve of **6** showed Cotton effect at λ_max_ (Δε) 293 (+13.1), 249 (–4.7), 211 (+6.8) nm, matching with the calculated ECD curve of (3*S*)-**6** and opposite to that of (3*R*)-**6** (Figure 5). Thus, the absolute configuration of **6** was unambiguous determined as *S*-, that is (*S*)-4-benzyl-3-oxo-3,4-dihydro-1*H*-pyrrolo[2,1-*c*][1,4]oxazine-6-carbaldehyde.

Compounds **3**–**5** were found to have the molecular formulas of C_12_H_15_NO_3_, C_11_H_13_NO_3_, and C_12_H_15_NO_3_ by HRESI-MS peaks at *m*/*z* 222.1131 [M + H]^+^, 208.0974 [M + H]^+^, and 222.1129 [M + H]^+^(calcd. for C_12_H_16_NO_3_ 222.1125, C_11_H_14_NO_3_ 208.0968, C_12_H_16_NO_3_ 222.1125), respectively. The similarity of ultraviolet (UV) and NMR spectra between **3**–**5** and **6** indicates compounds **3**–**5** contain the same3-oxo-3,4-dihydro-1*H*-pyrrolo[2,1-*c*][1,4]oxazine-6-carbaldehyde nucleus. The remaining moieties of C_4_H_9_, C_3_H_7_ and C_4_H_9_ for **3**–**5** could be deduced as isobutyl, isopropyl, and *sec*-butyl, respectively, from their 1D NMR data (Table 1). Thus, the planar structures of **3**–**5** were 4-isobutyl-3-oxo-3,4-dihydro-1*H*-pyrrolo[2,1-*c*][1,4]oxazine-6-carbaldehyde [8], 4-isopropyl-3-oxo-3,4-dihydro-1*H*-pyrrolo[2,1-*c*][1,4]oxazine-6-carbaldehyde [8], and 4-(2-methylbutyl)-3-oxo-3,4-dihydro-1*H*-pyrrolo [2,1-*c*] [1,4]oxazine-6-carbaldehyde [8,9], respectively. The planar structure **3** was further supported by ^1^H-^1^H COSY correlations of H-14/H-13/H-12/H-11/H-3 and H-6 (δ_H_ 7.14) to H-7 (δ_H_ 6.31) combined with the key HMBC correlations from H-11 (δ_H_ 2.00, 1.60) to C-2 (δ_C_ 167.5), C-3 (δ_C_ 56.4), C-13 (δ_C_ 21.6), and C-14 (δ_C_ 23.3), from H-9 (δ_H_ 5.70, 5.51) to C-2, C-7 (δ_C_ 107.2), and C-8 (δ_C_ 132.5), from H-3 (δ_H_ 5.65) to C-2, C-8, C-12 (δ_C_ 24.7), of H-7 (δ_H_ 6.31) to C-5 (δ_C_ 130.7), C-6 (δ_C_ 125.2), of H-6 to C-7, C-8, and C-10 (δ_C_ 180.1). The same lactone sector rule and ECD quantum chemical calculation used in **6** were also applied to elucidate the absolute configurations of **3**–**5**. Thus, the same (3*S*)-configurations of **3**–**5** could be deduced from the same negative sign of the specific rotations and the positive CD Cotton effects at the long wave length along with concordance of CD with the calculated ECD of (3*S*)-isomer and opposition to ECD of (3*R*)-isomer (Figure 5).

**Table 1:** ^1^H and ^13^C NMR data for **3**–**6** (δ in ppm).

Position	3		4		5		6	
	δ_C_, Type	δ_H_, Mult. (*J* in Hz)	δ_C_, Type	δ_H_, Mult. (*J* in Hz)	δ_C_, Type	δ_H_, Mult. (*J* in Hz)	δ_C_, Type	δ_H_, Mult. (*J* in Hz)
2	167.5, qC		166.7, qC		166.5, qC		167.2, qC	
3	56.4, CH	5.65, dd, (5.7, 10.2)	63.1, CH	5.36, d, (8.0)	62.0, CH	5.46, d, (6.9)	59.0, CH	5.84, t, (5.6)
5	130.7, qC		131.4, qC		131.2, qC		130.7, qC	
6	125.2, CH	7.14, d, (4.0)	125.4, CH	7.17, d, (4.0)	125.6, CH	7.2, d, (3.8)	125.4, CH	7.19, d, (4.0)
7	107.2, CH	6.31, d, (4.0)	107.2, CH	6.34, d, (4.0)	107.1, CH	6.34, d, (3.8)	106.4, CH	6.17, d, (4.0)
8	132.5, qC		132.7, qC		132.8, qC		132.4, qC	
9	63.7, CH_2_	5.70, d, (15.2)	64.2, CH_2_	5.64, d, (15.5)	64.4, CH_2_	5.64, d, (15.6)	63.6, CH_2_	5.24, d, (15.1)
		5.51, d, (15.2)		5.53, d, (15.5)		5.54, d, (15.6)		4.06, d, (15.1)
10	180.1, CH	9.49, s	180.2, CH	9.51, s	180.0, CH	9.5, s	179.9, CH	9.51, s
11	41.3, CH_2_	2.00, ddd, (4.3, 10.2, 13.4)	32.4, CH	2.33, m	39.3, CH	2.08, m	39.5, CH_2_	3.33, m
		1.60, ddd, (5.7, 9.3, 13.4)						
12	24.7, CH	1.66, m	19.3, CH_3_	0.97, d, (6.8)	25.8, CH_2_	1.39, m; 1.25, m	135.1, qC	
13	21.6, CH_3_	1.00, d, (6.4)	18.8, CH_3_	0.92, d, (6.8)	15.5, CH_3_	0.90, d, (7.0)	129.8, CH	6.85, d, (7.3)
14	23.3, CH_3_	0.89, d, (6.4)			11.8, CH_3_	0.91, t, (7.1)	129.1, CH	7.25, t, (7.3)
15							128.2, CH	7.29, t, (7.3)
16							129.1, CH	7.25, t, (7.3)
17							129.8, CH	6.85, d, (7.3)

### 2.2. The Bioactivities of Compounds **1**–**13** from *J. endophytica* 161111

Compounds **1**–**13** were tested for antivirus effects on H1N1 by the cytopathic effect (CPE) inhibition assay [45,46] and ribavirin was used as the positive control with an *IC_50_* value of 23.1 ± 1.7 μg/mL. The results (Table S1 in Supplementary Information) showed that compounds **8**–**11** showed moderate anti-H1N1 activity with *IC_50_* values of 38.3 ± 1.2, 25.0 ± 3.6, 39.7 ± 5.6, and 45.9 ± 2.1 μg/mL, respectively. In addition, the cytotoxic effects of **8**–**11** on Madin-Daby canine kidney (MDCK) normal cells were also evaluated by 3-(4,5-dimethyl-2-thiazolyl)-2,5-diphenyl-2*H*-tetrazolium bromide (MTT) [47]. The results showed that compounds **8**–**11** and ribavirin exhibited very weak cytotoxicities against MDCK normal cell with the half maximal cytotoxic concentration (*CC_50_*) values of 116.3 ± 12.1, 403.2 ± 31.4, 124.1 ± 10.5, 522.5 ± 24.5, and 744.2 ± 18.5 μg/mL, respectively. The *SI* values of compounds **8**–**11** and ribavirin were 3.0, 16.1, 3.1, 11.4, and 32.2, respectively.

Both naturally occurring and chemically synthesized β-carbolines exhibited good activities against a set of virus, such as human immunodeficiency virus (HIV) [26,48], herpes simplex virus (HSV) [27,49], vaccinia virus [50], vesicular stomatitis virus [50,51], poliovirus [49], and influenza A and B virus [51]. It was reported that the derivatives with the oxathiazepine 7-membered ring showed potential activities against influenza A and B [51]. This study showed that the simple β-carboline alkaloids were active against H1N1 virus and the unsubstituted H-3 is essential for the anti-H1N1 activity of this kind of compounds. In addition, the compound **9** could be a promising new hit for anti-H1N1 drugs. In addition, further chemical modifications are necessary to improve the anti-H1N1 virus activity.

## 3. Experimental Section

### 3.1. General Experimental Procedures

Specific rotations were obtained on a JASCO P-1020 digital polarimeter. UV spectra were measured on a Beckman DU 640 spectrophotometer. IR spectra were recorded on a Nicolet Nexus 470 spectrophotometer as KBr disks. CD spectra were collected using a JASCO J-715 spectropolarimeter. NMR data of **7**, **8**, **11**, and **13** were measured on a JEOL JNM-ECP 600 spectrometer, while NMR of **1**, **2**, **3**–**6**, **9**, **10**, and **12**, and all NOESY spectra were recorded on a Bruker Avance 600 spectrometer. Electrospray ionization mass spectra (ESIMS) and HRESIMS measurements were taken on a Q-TOFULTIMA GLOBAL GAA076 LC mass spectrometer. Semipreparative HPLC was performed using an octadecylsilyl (ODS) column (YMC-pak ODS-A, Allentown, PA, USA, 10 × 250 mm, 5 μm, 4.0 mL/min) and C_3_ column (Agilent Zorbax StableBond C_3_, Palo Alto, CA, USA, 4.6 × 150 mm, 5 μm, 1.0 mL/min). TLC and column chromatography (CC, 2.5 × 103 cm) were performed on plates pre-coated with silica gel GF_254_ (10–40 μm, Qingdao Marine Chemical Factory, Qingdao, China), and over Sephadex LH-20 (Amersham Biosciences, Uppsala, Sweden), respectively. Vacuum-liquid chromatography (VLC, 6 × 24 cm and 3.6 × 30 cm) utilized silica gel (200–300 mesh, Qingdao Marine Chemical Factory, Qingdao, China) and RP-18 (40–63 μm, Merck, Darmstadt, Germany). Glucose (Shanghai Huixing Biochemical Reagent Co., Ltd., Shanghai, China); yeast extract and peptone (Beinjing Shuangxuan Microbe Culture Medium Products Factory, Beijing, China); CaCO_3_ (Tianjijn Bodi Chemical Co., Ltd., Tianjin, China); KNO_3_ (Sinopharm Chemical Reagent Co., Ltd., Shanghai, China); NaCl (Sinopharm Chemical Reagent Co., Ltd., Shanghai, China).

### 3.2. Actinomycetes Material

*Jishengella endophytica* 161111 was isolated and identified from the healthy roots of the mangrove plant, *Xylocarpus granatum*, collected from the mangrove reserve zone in Hainan Province, China [5]. The producing strain was prepared on oatmeal agar (ISP3) medium and stored in Hong’s Lab. at 4 °C.

### 3.3. Fermentation and Extraction

The spores of *J. endophytica* 161111 were directly cultured in 1000 mL Erlenmeyer flasks containing 200 mL fermentation media consisted of glucose 2%, yeast extract 0.5%, peptone 0.5%, KNO_3_ 1.5%, CaCO_3_ 0.4%, and NaCl 0.4% (pH 7.2). The cultures were incubated on a rotatory shaker at 220 rpm at 28 °C for 30 days. The whole fermentation broth (100 L) was divided into three equal parts and extracted three times with equal volumes of EtOAc separately. The whole EtOAc solutions were combined and evaporated under reduced pressure to give a dark brown gum (10 g).

### 3.4. Purification and Identification

The EtOAc extract (10 g) was subjected to SiO_2_ VLC, eluting with CH_2_Cl_2_–petroleum ether (0%~100%) and then with MeOH–CH_2_Cl_2_ (0%~50%), to give nine fractions (Fr.1–Fr.9). Fraction 3 (1.31 g) was separated into three subfractions by CC over Sephadex LH-20, eluted with MeOH/CH_2_Cl_2_ (1:1). Fraction 3-3 (351 mg) was further subjected to HPLC separation eluteding with 50% MeCN/H_2_O to yield **3** (2 mg, *t*_R_ 9.93 min) and **6** (1 mg, *t*_R_ 10.74 min). Compound **7** (25 mg) was purified from the precipitate of fraction 5 (512 mg) after washing with MeOH, and the filtrate of fraction 5 was separated into five parts (Fr.5-1–Fr.5-4) by Sephadex LH-20 with CH_3_OH. Compound **11** (3 mg, *t*_R_ 6.46 min) was obtained from Fr.5-4 (71 mg) by HPLC purification eluting with 60% MeOH. Fraction 4 (413 mg) was separated by CC over RP-18 to afford four subfractions (Fr.4-1–Fr.4-5), and compounds **9** (3 mg, *t*_R_ 7.3 min) and **10** (3 mg, *t*_R_ 9.7 min) were obtained from Fr.4-4 (93 mg) by HPLC purifications eluted with 60% MeOH and 50% MeOH, respectively. Fr.4-2 (87 mg) and Fr.4-3 (77 mg) were both further separated into four parts (Fr.4-2-1–Fr.4-2-4 and Fr.4-3-1–Fr.4-3-4) by Sephadex LH-20 with MeOH. Compounds **12** (4 mg, *t*_R_ 8.97 min) and **13** (5 mg, *t*_R_ 7.6 min) were purified from Fr.4-2-2 (21 mg) and Fr.4-2-3 (17 mg) by HPLC eluting with 40% and 60% MeOH, respectively. And Fr.4-3-2 (16 mg) and Fr.4-3-3 (10 mg) were purified by HPLC eluting with 70% MeOH to give **4** (2 mg, *t*_R_ 5.3 min) and **5** (2 mg, *t*_R_ 5.0 min), respectively. Fraction **7** (414 mg) gave four parts (Fr.7-1–Fr.7-4) after subjection to CC over Sephadex LH-20 with MeOH. Fr.7-2 (92 mg) was further purified by HPLC, with 30% MeOH, to yield the mixture (3 mg, *t*_R_ 12.33 min) of **1** and **2** by ODS column. The mixture was further purified by HPLC over C_3_ column with 25% MeOH to give pure **1** (2 mg, *t*_R_ 10.08 min) and **2** (1 mg, *t*_R_ 11.98 min). Compound **8** (7 mg, *t*_R_ 8.68 min) was obtained from Fr.7-3 (133 mg) by HPLC with 50% MeOH.

**2-(Furan-2-yl)-6-(2*S*,3*S*,4-trihydroxybutyl)pyrazine (1)**: A brown oil. [α]_D_^20^ −11.5 (*c* 0.05, MeOH); UV (MeOH) λ_max_ (log ε) 240 (3.4), 273 (3.6), 328 (3.4) nm; CD (*c* 0.05, MeOH) λ_ext_ (Δε) 310 (−0.14), 284 (+0.12), 237 (−1.3) nm; ^1^H (600 MHz, MeOH-*d*_4_) and ^13^C NMR (150 MHz, MeOH-*d*_4_), see Table 2; HRESIMS *m*/*z* 273.0849 [M + Na]^+^ (calcd for C_12_H_14_N_2_O_4_Na 273.0846).

**Table 2:** ^1^H and ^13^C NMR data for **1** and **2** (δ in ppm).

Position	1			2		
	δ_C_, Type	δ_H_, Mult. (*J* in Hz)	δ_H_, Mult. (*J* in Hz, −4 °C)	δ_C_, Type	δ_H_, Mult. (*J* in Hz)	δ_H_, Mult. (*J* in Hz, −4 °C)
2	144.2, qC			142.5, qC		
3	136.7, CH	8.78, s	8.79, s	138.7, CH	8.90, d, (0.7)	8.91, d, (1.0)
5	142.7, CH	8.38, s	8.38, s	153.4, qC		
6	155.3, qC			144.3, CH	8.50, d, (0.7)	8.50, d, (1.0)
2′	151.1, qC			151.0, qC		
3′	110.5, CH	7.23, d, (4.0)	7.25, d, 3.4	109.9, CH	7.18, d, (3.4)	7.19,d, (3.4)
4′	112.0, CH	6.63, dd, (2.1, 4.0)	6.64, dd, (1.7, 3.4)	111.9, CH	6.63, d, (1.7, 3.4)	6.64, dd, (1.7, 3.4)
5′	144.6, CH	7.72, d, (2.1)	7.74, d, (1.7)	144.6, CH	7.71, d, (1.7)	7.73, d, (1.7)
1″	38.4, CH_2_	3.24, dd, (3.1, 14.0)	3.24, dd, (2.9, 14.0)	38.3, CH_2_	3.22, dd, (2.8, 14.0)	3.22, dd, (2.8, 14.0)
		2.93, dd, (9.4, 14.0)	2.91, dd, (9.7, 14.0)		2.93, dd, (9.4, 14.0)	2.91, dd, (9.5, 14.1)
2″	71.5, CH	4.03, ddd, (3.1, 7.0, 9.4)	4.01, ddd, (2.9, 7.2, 9.7)	71.6, CH	3.97, ddd, (2.8, 7.0, 9.4)	3.95, ddd, (2.8, 7.1, 9.5)
3″	74.8, CH	3.58, ddd, (3.8, 7.0, 6.2)	3.57, ddd, (3.8, 7.2, 6.5)	74.8, CH	3.57, (3.7, 7.0, 6.5)	3.56, ddd, (3.7, 7.1, 6.5)
4″	63.2, CH_2_	3.80, dd, (3.8, 11.4)	3.80, dd, (3.8, 11.2)	63.2, CH_2_	3.79, dd, (3.7, 11.3)	3.78, dd, (3.7, 11.3)
		3.65, dd, (6.2, 11.4)	3.63, dd, (6.5, 11.2)		3.64, dd, (6.2, 11.3)	3.62, dd, (6.5, 11.3)

**2-(Furan-2-yl)-5-(2*S*,3*S*,4-trihydroxybutyl)pyrazine (2)**: Brown oil. [α]_D_^20^ −11.3 (*c* 0.03, MeOH); UV (MeOH) λ_max_ (log ε) 240 (3.2), 273 (3.4), and 328 (3.1) nm; CD (*c* 0.05, MeOH) λ_ext_ (Δε) 316 (−0.54), 267 (+0.35), 235 (−1.0) nm; ^1^H (600 MHz, MeOH-*d*_4_) and ^13^C NMR (150 MHz, MeOH-*d*_4_), see Table 2; HRESIMS *m*/*z* 273.0849 [M + Na]^+^ (calcd for C_12_H_1__4_N_2_O_4_Na 273.0846).

**(*S*)-4-Isobutyl-3-oxo-3,4-dihydro-1*H*-pyrrolo[2,1-*c*][1,4]oxazine-6-carbaldehyde (3)**: A brown oil. [α]_D_^20^ −32 (*c* 0.05, acetone); UV (MeOH) λ_max_ (log ε) 254 (3.3), and 291 (3.9) nm; CD (*c* 0.08, MeOH) λ_max_ (Δε) 292 (+2.9), 248 (−1.0), 207 (+2.5) nm; ^1^H (600 MHz, DMSO-*d*_6_) and ^13^C NMR (150 MHz, DMSO-*d*_6_), see Table 1; HRESIMS *m*/*z* 222.1131 [M + H]^+^ (calcd for C_12_H_14_N_2_O_4_Na 222.1125).

**(*S*)-4-Isopropyl-3-oxo-3,4-dihydro-1*H*-pyrrolo[2,1-*c*][1,4]oxazine-6-carbaldehyde (4)**: A brown oil. [α]_D_^20^ −88 (*c* 0.12, acetone); UV (MeOH) λ_max_ (log ε) 254 (3.4) and 291 (4.03) nm; CD (*c* 0.02, MeOH) λ_max_ (Δε) 298 (+16.1), 290 (−1.9), 280 (+6.6), 253 (−8.9), 208 (+8.8) nm; ^1^H (600 MHz, DMSO-*d*_6_) and ^13^C NMR (150 MHz, DMSO-*d*_6_), see Table 1; HRESIMS *m*/*z* 208.0974 [M + H]^+^ (calcd for C_12_H_1__4_N_2_O_4_Na 208.0968).

**(4*S*)-4-(2-Methylbutyl)-3-oxo-3,4-dihydro-1*H*-pyrrolo[2,1-*c*][1,4]oxazine-6-carbaldehyde (5)**: A brown oil; [α]_D_^20^ −57 (*c* 0.06, acetone); UV (MeOH) λ_max_ (log ε) 254 (3.6) and 291 (4.2) nm; CD (*c* 0.08, MeOH) λ_max_ (Δε) 293 (+5.8), 252 (−2.7), 208 (+3.1) nm; ^1^H (600 MHz, DMSO-*d*_6_) and ^13^C NMR (150 MHz, DMSO-*d*_6_), see Table 1; HRESIMS *m*/*z* 222.1129 [M + H]^+^ (calcd for C_12_H_1__4_N_2_O_4_Na 222.1125).

**(*S*)-4-Benzyl-3-oxo-3,4-dihydro-1*H*-pyrrolo[2,1-*c*][1,4]oxazine-6-carbaldehyde (6)**: A brown oil. [α]_D_^20^ −46 (*c* 0.1, acetone); UV (MeOH) λ_max_ (log ε) 257 (3.4) and 295 (3.8) nm; CD (*c* 0.02, MeOH) λ_max_ (Δε) 293 (+13.1), 249 (−4.7), 211 (+6.8) nm; ^1^H (600 MHz, DMSO-*d*_6_) and ^13^C NMR (150 MHz, DMSO-*d*_6_) see Table 1; HRESIMS *m*/*z* 256.0976 [M + H]^+^ (calcd for C_12_H_1__4_N_2_O_4_Na 256.0968).

### 3.5. Bioassays

The antiviral activity against H1N1 virus was evaluated by the CPE inhibition assay [45,46]. Confluent MDCK cell monolayers were firstly incubated with influenza virus (A/Puerto Rico/8/34 (H1N1), PR/8) at 37 °C for 1 h. After removing the virus dilution, cells were maintained in infecting media (RPMI 1640, 4 μg/mL of trypsin) containing different concentrations of test compounds and ribavirin (positive control) at 37 °C. After 48 h incubation at 37 °C, the cells were fixed with 100 μL of 4% formaldehyde for 20 min at room temperature. After removal of the formaldehyde, the cells were stained with 0.1% crystal violet for 30 min. The plates were washed and dried, and the intensity of crystal violet staining for each well was measured in a microplate reader (Bio-Rad, Hercules, CA, USA) at 570 nm. The *IC_50_* was calculated as the compound concentration required inhibiting influenza virus yield at 48 h post-infection by 50%. Ribavirin was used as the positive control with an *IC_50_* value of 23.1 ± 1.7 μg/mL.

Cytotoxicity was assayed by the MTT [47]. In the MTT assay, MDCK normal cell line were cultured in RPMI-1640, supplemented with 10% FBS, under a humidified atmosphere of 5% CO_2_ and 95% air at 37 °C, cell suspensions with a density of 4.6 × 10^4^ cells/mL (198 μL) was plated in 96-well microtiter plates and incubated for 24 h. Then, the test solutions in MeOH (2 μL) were added to each well and further incubated for 36 h. The MTT solution (20 µL, 5 mg/mL in RPMI-1640 medium) was then added to each well and incubated for 4 h. Old medium containing MTT (150 μL) was then gently replaced by dimethylsulfoxide (DMSO) and pipetted to dissolve any formazan crystals formed. Absorbance was then determined on a Spectra Max Plus plate reader at 570 nm. Ribavirin was used as positive control (*CC_50_* 744.2 ± 18.5 μg/mL for MDCK normal cell line).

## 4. Conclusions

A new pyrazine derivative, 2-(furan-2-yl)-6-(2*S*,3*S*,4-trihydroxybutyl)pyrazine (**1**), was isolated and identified from the fermentation products of *Jishengella endophytica* 161111 endophytic with mangrove plant, *Xylocarpus granatum* (Meliaceae). The absolute configurations and the key reference data of [α]_D_, CD and ^13^C NMR of 2-(furan-2-yl)-5-(2*S*,3*S*,4-trihydroxybutyl)pyrazine (**2**), (*S*)-4-isobutyl-3-oxo-3,4-dihydro-1*H*-pyrrolo[2,1-*c*][1,4]oxazine-6-carbaldehyde (**3**), (*S*)-4-isopropyl-3-oxo-3,4-dihydro-1*H*-pyrrolo[2,1-*c*][1,4]oxazine-6-carbaldehyde (**4**), and (4*S*)-4-(2-methylbutyl)-3-oxo-3,4-dihydro-1*H*-pyrrolo[2,1-*c*][1,4]oxazine-6-carbaldehyde (**5**) were reported here for the first time. In addition, β-carboline alkaloid **9** exhibited moderate anti-H1N1 virus activity and weak cytotoxicity.

## Supplementary Files

Supplementary File 1 Supplementary Information (PDF, 3531 KB)
