# Supplementary material for: Alkaloids from the Mangrove-Derived Actinomycete Jishengella endophytica 161111
Source: Mar Drugs. 2014 Jan 21;12(1):477–90. doi: 10.3390/md12010477 (PMC3917282; doi:10.3390/md12010477)
Supplement: Supplementary File 1 — Supplementary Information (PDF, 3531 KB) [file marinedrugs-12-00477-s001.pdf]

Supplementary Information

S1. The Physicochemical Data of Compounds **7**–**13**.
Figure S1. The ^{1}H NMR (600 MHz, MeOH-d_4) spectrum of compound **1**.
Figure S2. The ^{1}H NMR (600 MHz, MeOH-d_4) spectrum of compound **1** at −4 ℃.
Figure S3. The DEPTQ (150 MHz, MeOH-d_4) spectrum of compound **1**.
Figure S4. The ^{1}H-^{1}H COSY (600 MHz, MeOH-d_4) spectrum of compound **1**.
Figure S5. The HSQC (150 MHz, MeOH-d_4) spectrum of compound **1**.
Figure S6. The HMBC (150 MHz, MeOH-d_4) spectrum of compound **1**.
Figure S7. The NOESY (600 MHz, MeOH-d_4) spectrum of compound **1**.
Figure S8. The NOESY (600 MHz, MeOH-d_4) spectrum of compound **1** at −4 ℃.
Figure S9. The ^{1}H NMR (600 MHz, MeOH-d_4) spectrum of compound **2**.
Figure S10. The ^{1}H NMR (600 MHz, MeOH-d_4) spectrum of compound **2** at −4 ℃.
Figure S11. The DEPTQ (150 MHz, MeOH-d_4) spectrum of compound **2**.
Figure S12. The ^{1}H-^{1}H COSY (600 MHz, MeOH-d_4) spectrum of compound **2**.
Figure S13. The HSQC (150 MHz, MeOH-d_4) spectrum of compound **2**.
Figure S14. The HMBC (150 MHz, MeOH-d_4) spectrum of compound **2**.
Figure S15. The NOESY (600 MHz, MeOH-d_4) spectrum of compound **2**.
Figure S16. The NOESY (600 MHz, MeOH-d_4) spectrum of compound **2** at −4 ℃.
Figure S17. The ^{1}H NMR (600 MHz, DMSO-d_6) spectrum of compound **3**.
Figure S18. The ^{13}C NMR (150 MHz, DMSO-d_6) spectrum of compound **3**.
Figure S19. The DEPT (150 MHz, DMSO-d_6) spectrum of compound **3**.
Figure S20. The ^{1}H-^{1}H COSY (600 MHz, DMSO-d_6) spectrum of compound **3**.
Figure S21. The HSQC (150 MHz, DMSO-d_6) spectrum of compound **3**.
Figure S22. The HMBC (150 MHz, DMSO-d_6) spectrum of compound **3**.
Figure S23. The ^{1}H NMR ((600 MHz, DMSO-d_6) spectrum of compound **4**.
Figure S24. The ^{13}C NMR (150 MHz, DMSO-d_6) spectrum of compound **4**.
Figure S25. The DEPT (150 MHz, DMSO-d_6) spectrum of compound **4**.
Figure S26. The ^{1}H NMR (600 MHz, DMSO-d_6) spectrum of compound **5**.
Figure S27. The DEPTQ (150 MHz, DMSO-d_6) spectrum of compound **5**.
Figure S28. The ^{1}H NMR (150 MHz, DMSO-d_6) spectrum of compound **6**.
Figure S29. The ^{13}C NMR (600 MHz, DMSO-d_6) spectrum of compound **6**.
Figure S30. The DEPT (150 MHz, DMSO-d_6) spectrum of compound **6**.
Table S1. Anti-H1N1 virus activities of **1**–**13**.
Table S2. The 2D NMR data for compounds **1**–**3**.

S1. The Physicochemical Data of the Known Compounds 7–13

Compound 7: Yellow solid; ^{1}H NMR (600 MHz, DMSO-d_6): δ 8.83 (1H, s, H-4), 8.41 (1H, d, *J* = 7.6 Hz, H-5), 7.34 (1H, dd, *J* = 7.6, 7.8 Hz, H-6), 7.64 (1H, t, *J* = 7.8 Hz, H-7), 7.81 (1H, d, *J* = 7.8 Hz, H-8), 11.6 (1H, s, NH-9), 7.41 (1H, *J* = 3.2 Hz, H-3′), 6.62 (1H, *J* = 3.2 Hz, H-4′), 4.67 (2H, s, H-6′), 5.51 (1H, s, OH-6′).^{13}C NMR (150 MHz, DMSO-d_6): δ 133.0 (C_q, C-1), 137.6 (C_q, C-3), 116.3 (CH, C-4), 130.4 (C_q, C-4a), 121.5 (C_q, C-4b), 122.7 (CH, C-5), 121.1 (CH, C-6), 129.5 (CH, C-7), 113.4 (CH, C-8), 141.9 (C_q, C-8a), 132.5 (C_q, C-9a), 167.0 (C_q, C-10), 151.8 (C_q, C-2′), 111.7 (CH, C-3′), 109.9 (CH, C-4′), 157.8 (C_q, C-5′), 56.5 (CH_2, C-6′). ESIMS *m*/*z* 309.1 $[M + H]^+$.

Compound 8: Pale yellow solid; ^{1}H NMR (600 MHz, DMSO-d_6): δ 8.36 (1H, d, *J* = 5.2 Hz, H-3), 8.06 (1H, d, *J* = 5.2 Hz, H-4), 8.25 (1H, d, *J* = 7.9 Hz, H-5), 7.27 (1H, t, *J* = 7.5 Hz, H-6), 7.59 (1H, t, *J* = 7.6 Hz, H-7), 7.76 (1H, d, *J* = 8.2 Hz, H-8), 11.27 (1H, s, NH-9), 7.20 (1H, d, *J* = 3.2 Hz, H-3′), 6.58 (1H, d, *J* = 3.2 Hz, H-4′), 4.65 (2H, s, H-6′), 5.55 (1H, s, OH-6′). ^{13}C NMR (150 MHz, DMSO-d_6): δ 133.6 (C_q, C-1), 138.7 (CH, C-3), 114.3 (CH, C-4), 130.0 (C_q, C-4a), 121.1 (C_q, C-4b), 122.2 (CH, C-5), 120.3 (CH, C-6), 129.0 (CH, C-7), 113.0 (CH, C-8), 141.5 (C_q, C-8a), 131.0 (C_q, C-9a), 152.7 (CH, C-2′), 110.2 (CH, C-3′), 109.7 (CH, C-4′), 157.2 (C_q, C-5′), 56.5 (CH_2, C-6′). ESIMS *m*/*z* 265.1 $[M + H]^+$.

Compound 9: Yellow solid; ^{1}H NMR (600 MHz, DMSO-d_6): δ 7.08 (1H, t, *J* = 6.6 Hz, H-3), 6.99 (1H, d, *J* = 6.6 Hz, H-4), 8.02 (1H, d, *J* = 8.0 Hz, H-5), 7.41 (1H, td, *J* = 8.0, 1.0 Hz, H-6), 7.17 (1H, td, *J* = 8.0, 1.0 Hz, H-7), 7.52 (1H, d, *J* = 8.0 Hz, H-8), 11.90 (1H, s, NH-9). ^{13}C NMR (150 MHz, DMSO-d_6): δ 156.1 (C_q, C-1), 120.0 (CH, C-3), 100.2 (CH, C-4), 122.4 (C_q, C-4a), 124.7 (C_q, C-4b), 121.8 (CH, C-5), 126.7 (CH, C-6), 125.0 (CH, C-7), 112.9 (CH, C-8), 128.4 (C_q, C-8a), 139.4 (C_q, C-9a). ESIMS *m*/*z* 185.1 $[M + H]^+$.

Compound 10: Pale yellow solid; ^{1}H NMR (600 MHz, Pyridine-d_5): δ 13.91 (1H, s, NH-1), 14.14 (1H, s, H-3), 8.06 (1H, s, H-6), 7.87 (1H, s, H-9), 2.34 (3H, s, H-11), 2.28 (3H, s, H-12). ^{13}C NMR (150 MHz, Pyridine-d_5): δ 153.1 (C_q, C-2), 163.4 (C_q, C-4), 132.3 (C_q, C-4a), 140.4 (C_q, C-5a), 131.0 (CH, C-6), 146.1 (C_q, C-7), 141.1 (C_q, C-8), 128.4 (C_q, C-9), 144.3 (C_q, C-9a), 149.0 (C_q, C-10a), 21.7 (CH_3,C-11), 21.1 (CH_3, C-12). ESIMS *m*/*z* 243.1 $[M + H]^+$.

Compound 11: Pale yellow solid; ^{1}H NMR (600 MHz, DMSO-d_6): δ 12.14 (1H, s, NH-1), 8.22 (1H, s, H-2), 8.10 (1H, d, *J* = 7.7 Hz, H-4), 7.23 (1H, td, *J* = 7.7, 0.9 Hz, H-5), 7.27 (1H, td, *J* = 7.7, 0.9 Hz, H-6), 7.52 (1H, d, *J* = 7.7 Hz, H-7), 9.93 (1H, s, H-8). ^{13}C NMR (150 MHz, DMSO-d_6): δ 139.1 (CH, C-2), 118.7 (C_q, C-3), 124.6 (C_q, C-3a), 121.4 (CH, C-4), 122.8 (CH, C-5), 124.1 (CH, C-6), 113.0 (CH, C-7), 137.6 (CH, C-7a), 185.7 (CH, C-8). ESIMS *m*/*z* 146.1 $[M + H]^+$.

Compound 12: Yellow solid; ^{1}H NMR (600 MHz, DMSO-d_6): δ 11.93 (1H, s, NH-1), 8.32 (1H, s, H-2), 8.15 (1H, d, *J* = 7.2 Hz, H-4), 7.17 (1H, td, *J* = 7.2, 1.2 Hz, H-5), 7.22 (1H, td, *J* = 7.2, 1.2 Hz, H-6), 7.46 (1H, d, *J* = 7.2 Hz, H-7), 4.56 (2H, s, H-9). ^{13}C NMR (150 MHz, DMSO-d_6): δ 133.8 (CH, C-2), 113.7 (C_q, C-3), 125.9 (C_q, C-3a), 121.6 (CH, C-4), 122.3 (CH, C-5), 123.3 (CH, C-6), 112.7 (CH, C-7), 136.8 (C_q, C-7a), 194.9 (C_q, C-8), 65.7 (CH_2, C-9). ESIMS *m*/*z* 176.1 $[M + H]^+$.

Compound 13: Brown oil; ^{1}H NMR (600 MHz, DMSO-d_6): δ 12.13 (1H, s, NH-1), 6.92 (1H, d, J = 4 Hz, H-3), 6.21 (1H, d, J = 4 Hz, H-4), 9.41 (1H, s, H-6), 4.34 (2H, s, H_2-7), 3.22 (3H, s, H_3-8). ^{13}C NMR (150 MHz, DMSO-d_6): δ 133.2 (C_q, C-2), 121.5 (CH, C-3), 111.0 (CH, C-4), 138.4 (C_q, C-5), 179.6 (C_q, C-6), 66.5 (CH_2, C-7), 57.9 (CH_3, C-8). ESIMS m/z 138.1 $[M - H]^-$.

Figure S1. The ^{1}H NMR (600 MHz, MeOH-d_4) spectrum of compound **1**.

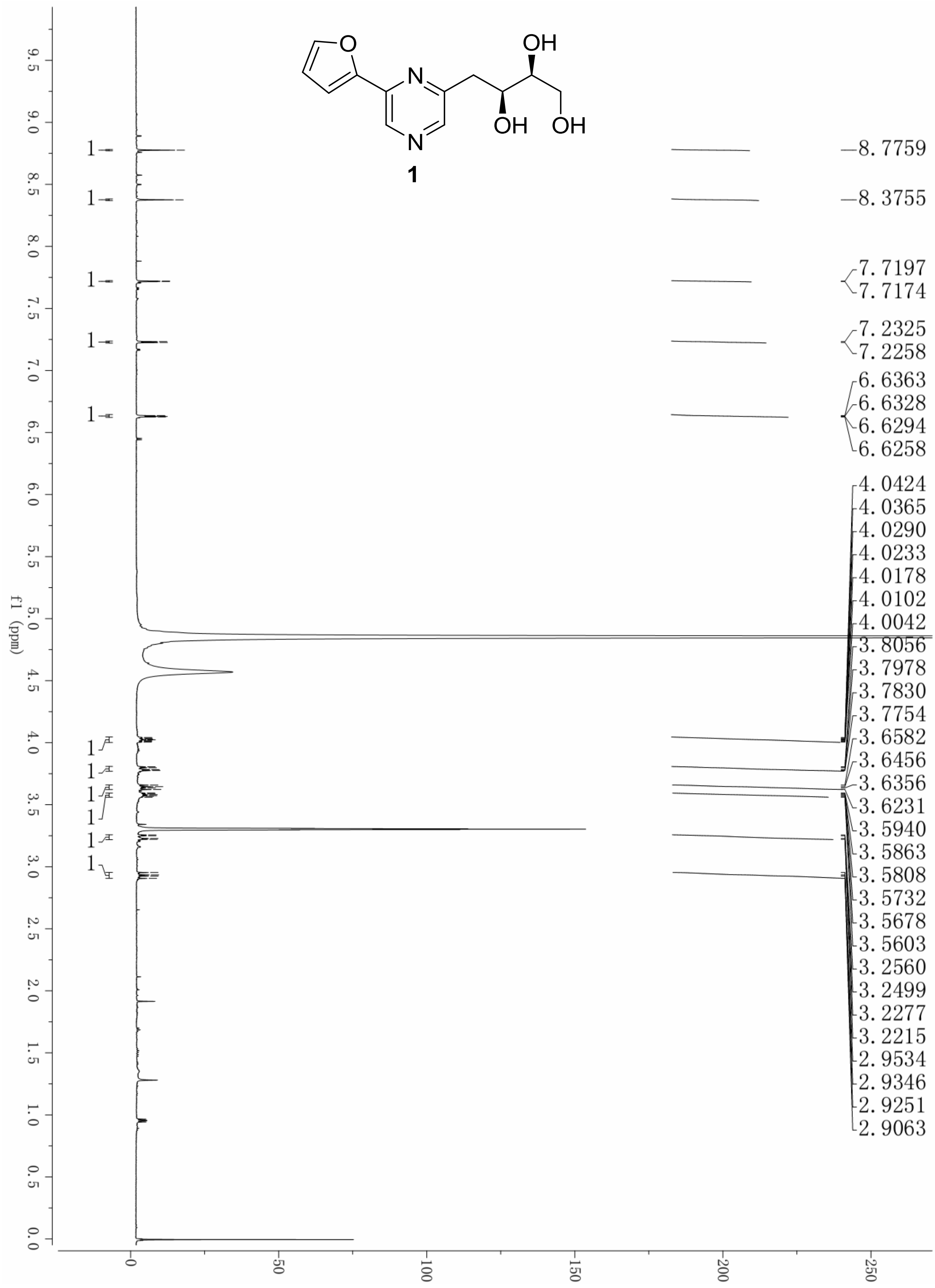

Figure S2. The ^{1}H NMR (600 MHz, MeOH-d_4) spectrum of compound **1** at −4 ℃.

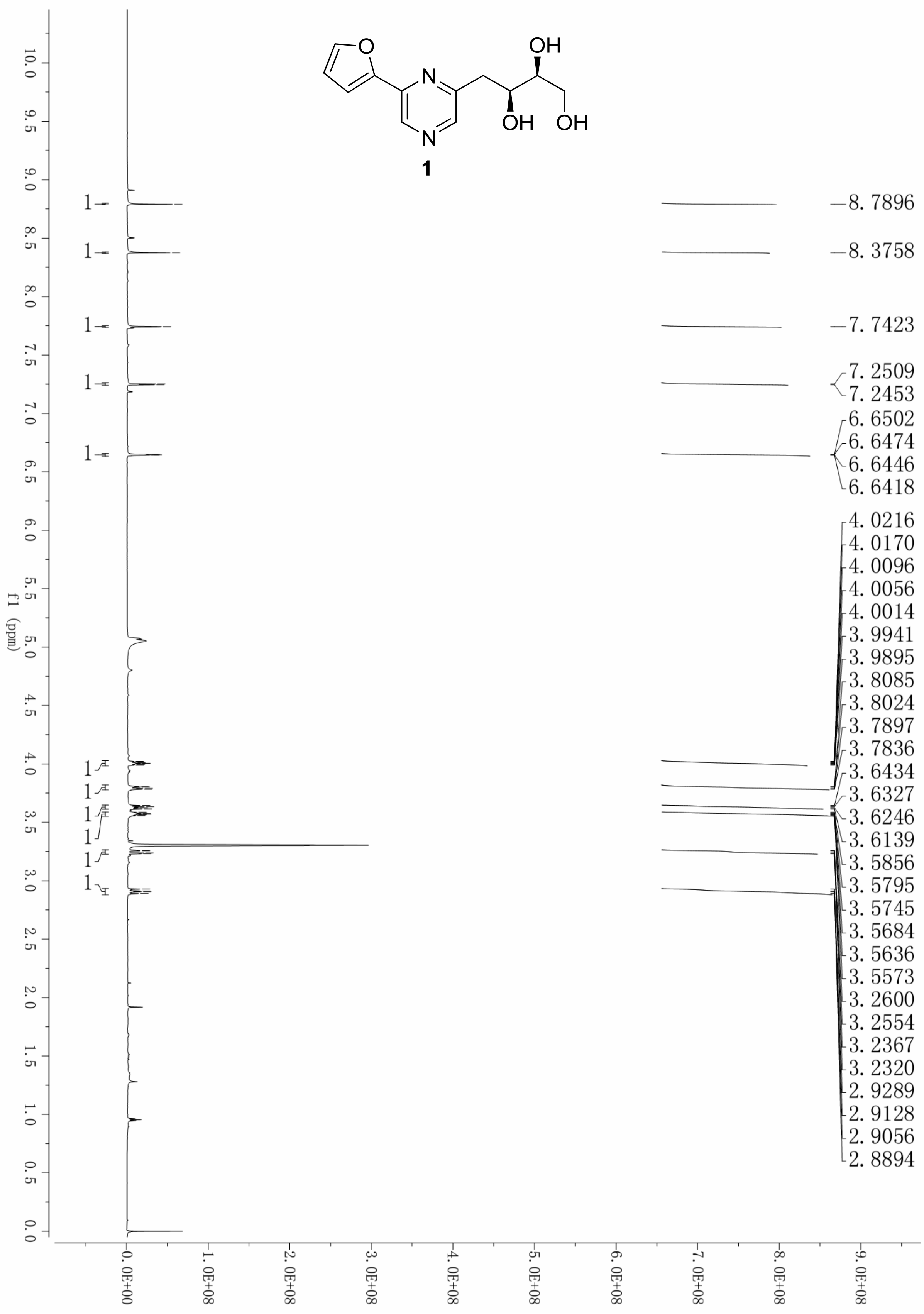

Figure S3. The DEPQ (150 MHz, MeOH-d_4) spectrum of compound **1**.

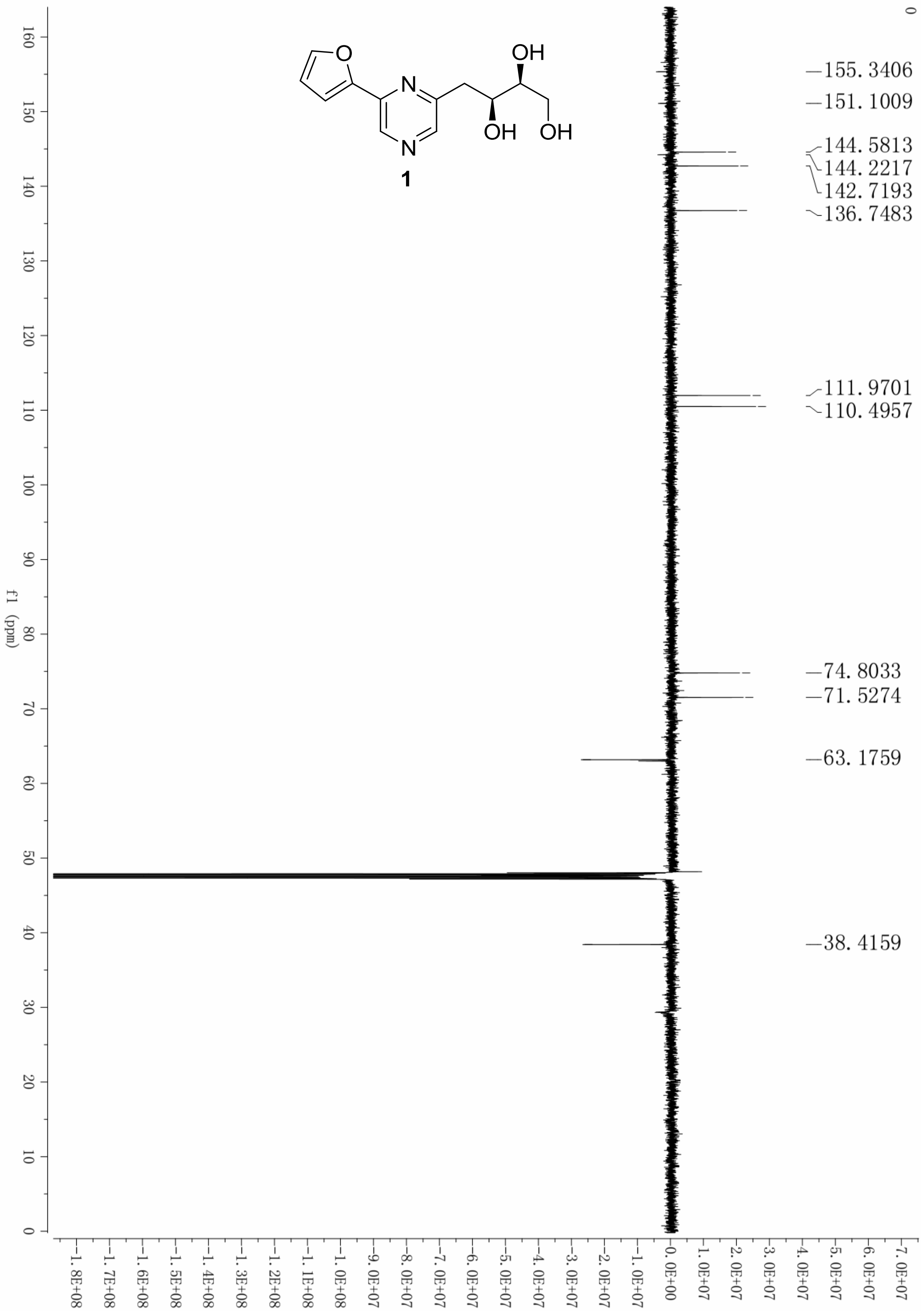

Figure S4. The ^{1}H-^{1}H COSY (600 MHz, MeOH-d_4) spectrum of compound **1**.

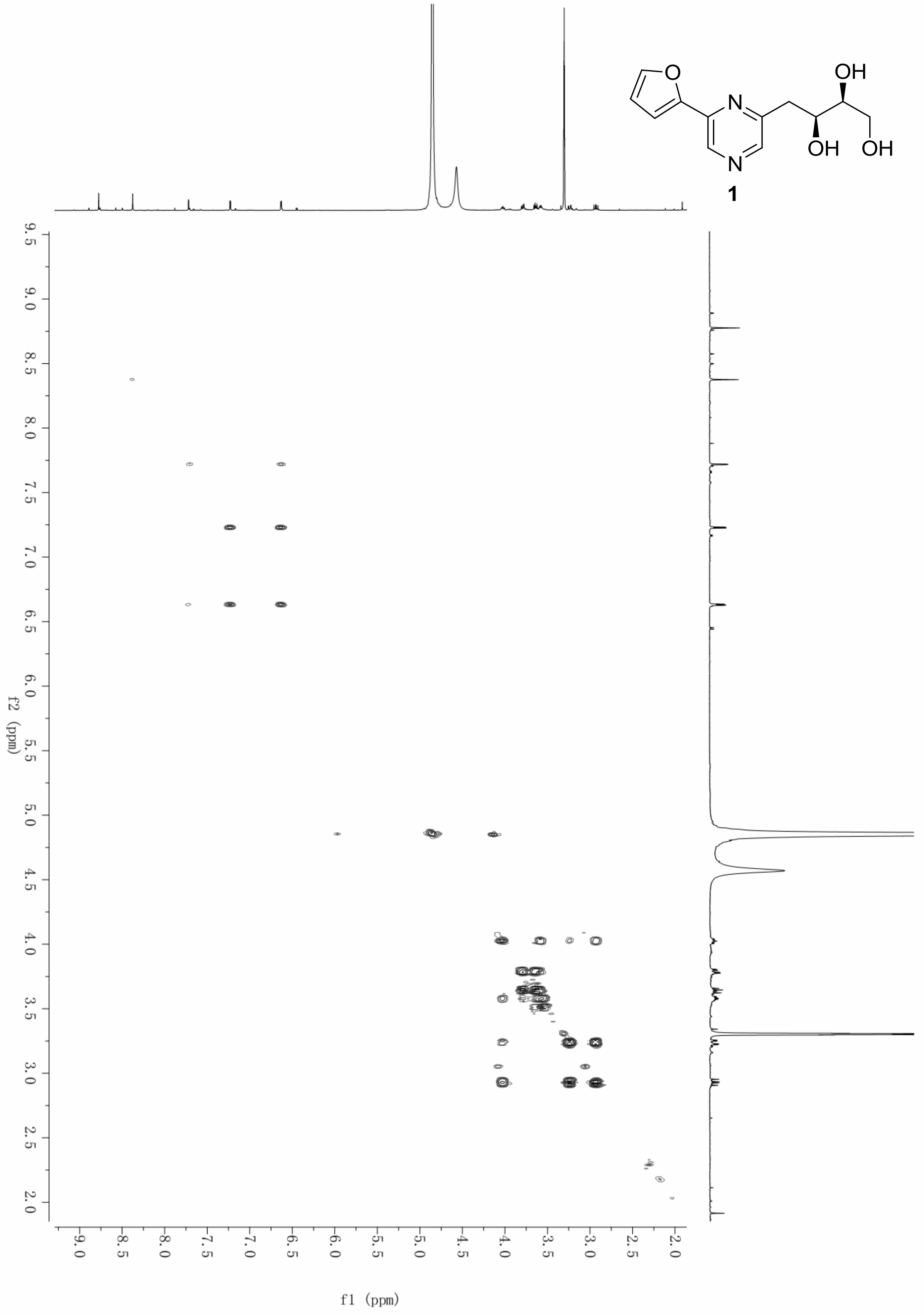

Figure S5. The HSQC (150 MHz, MeOH-d_4) spectrum of compound **1**.

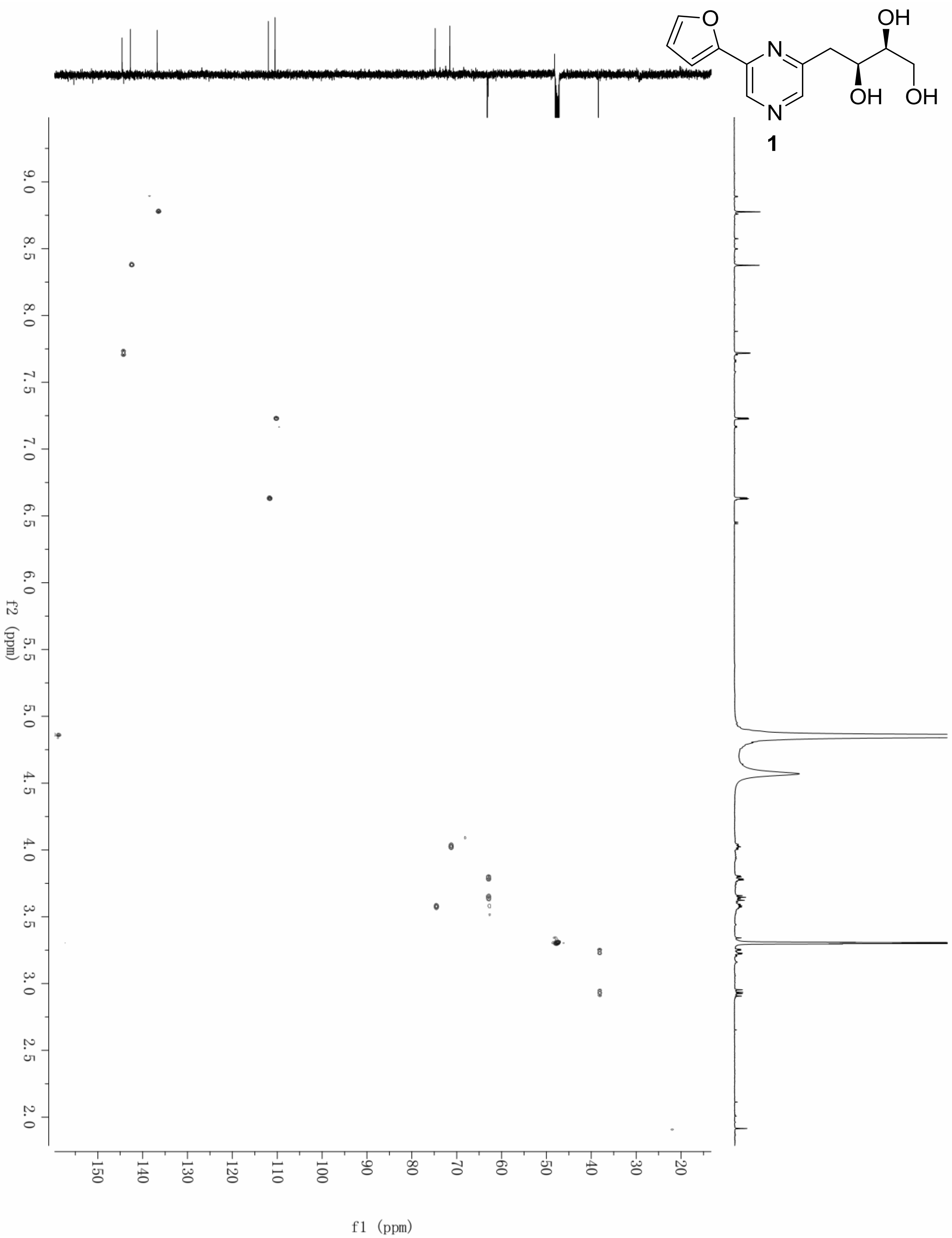

Figure S6. The HMBC (150 MHz, MeOH-d_4) spectrum of compound **1**.

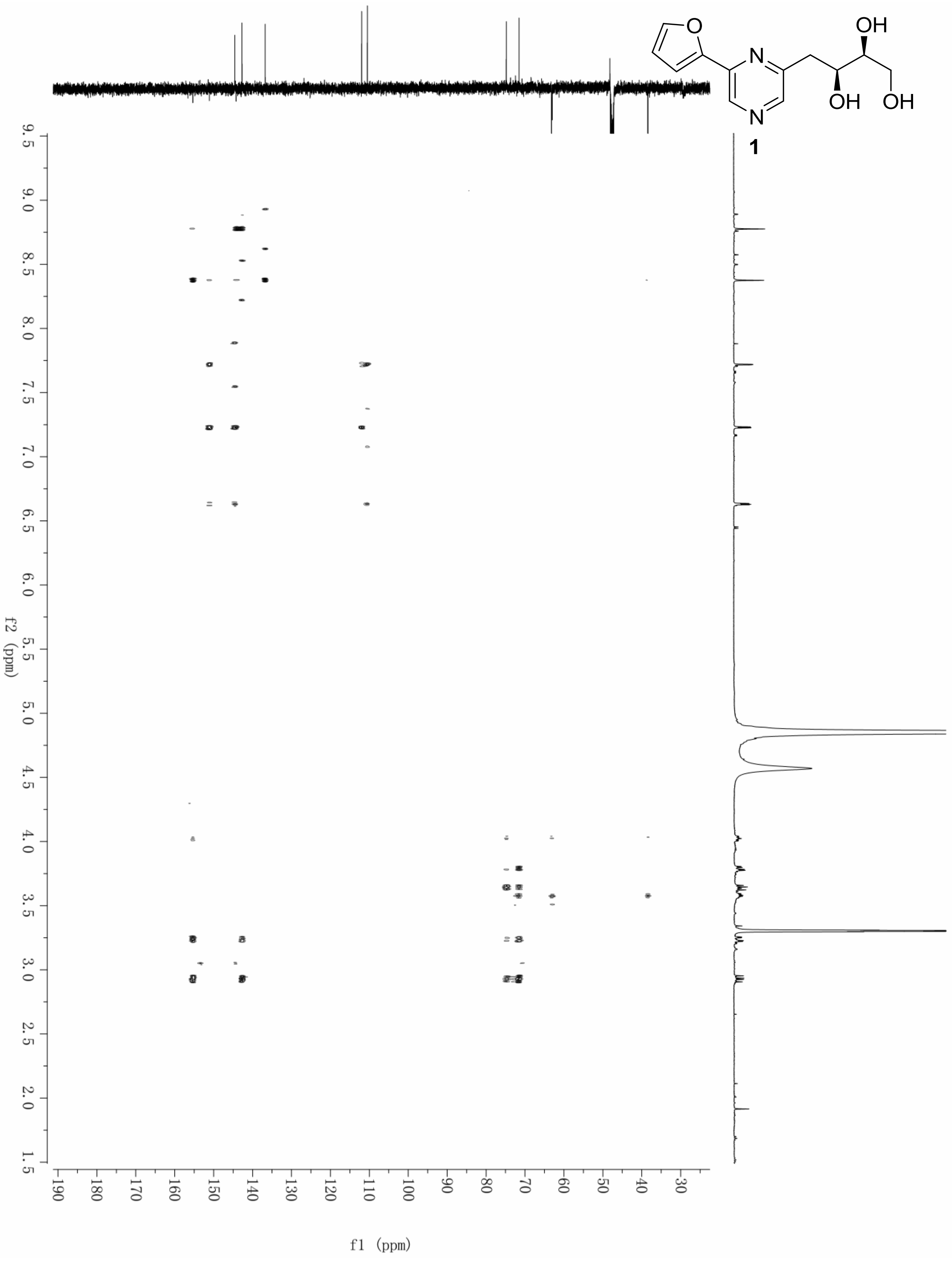

Figure S7. The NOESY (600 MHz, MeOH-d_4) spectrum of compound **1**.

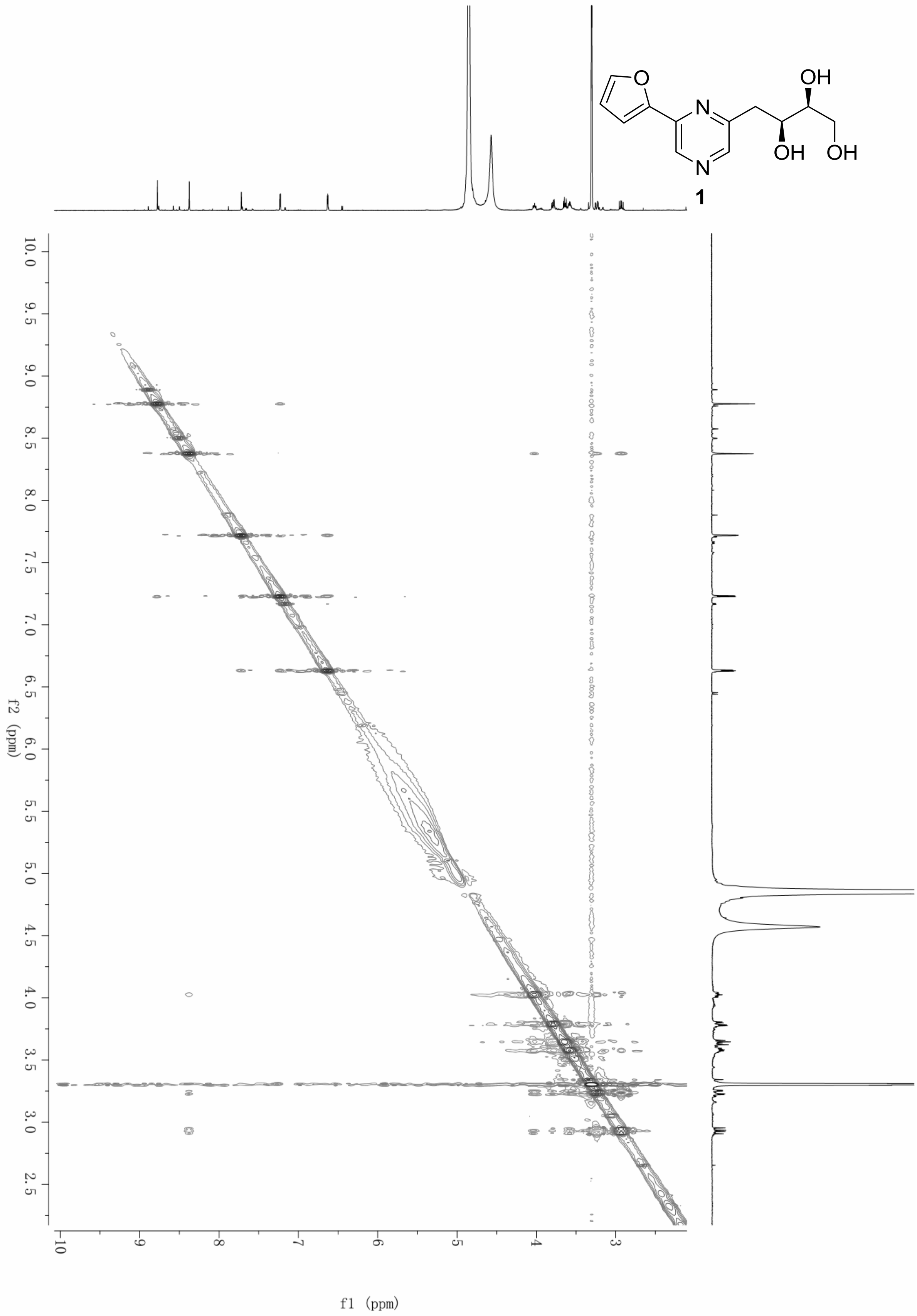

Figure S8. The NOESY (600 MHz, MeOH-d_4) spectrum of compound **1** at −4 ℃.

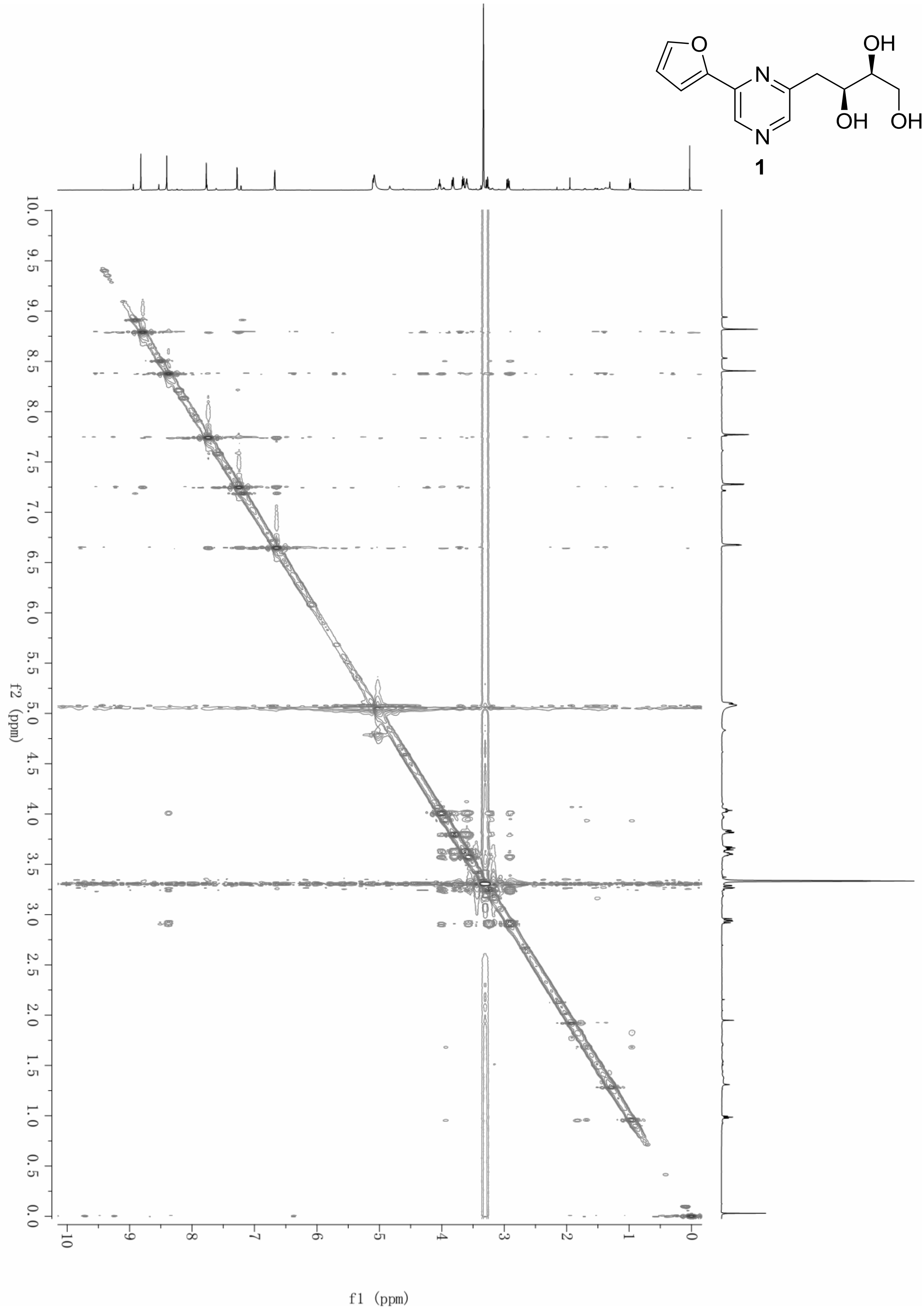

Figure S9. The ^{1}H NMR (600 MHz, MeOH-d_4) spectrum of compound **2**.

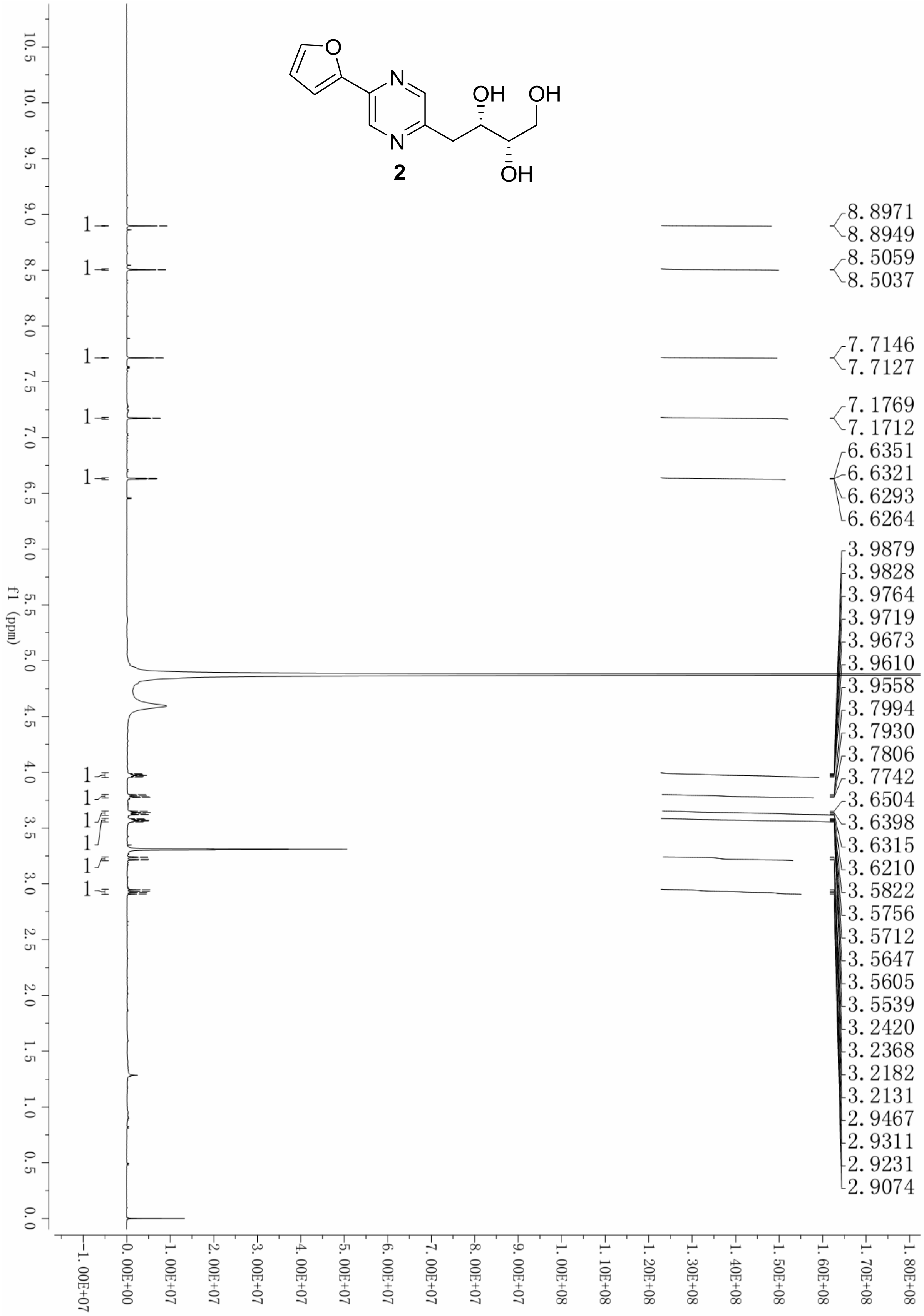

Figure S10. The ^{1}H NMR (600 MHz, MeOH-d_4) spectrum of compound **2** at −4 ℃.

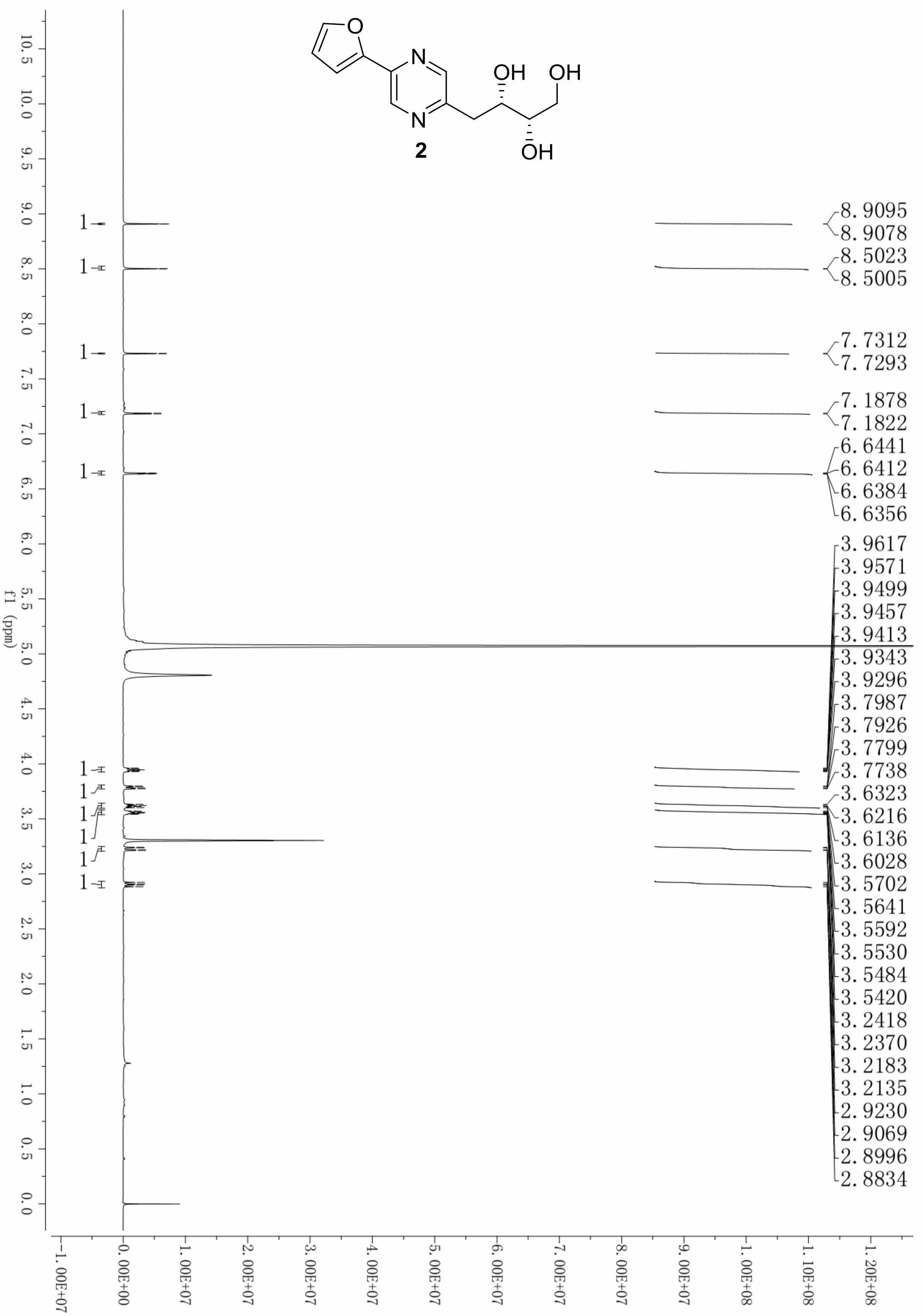

Figure S11. The DEPTQ (150 MHz, MeOH-d_4) spectrum of compound **2**.

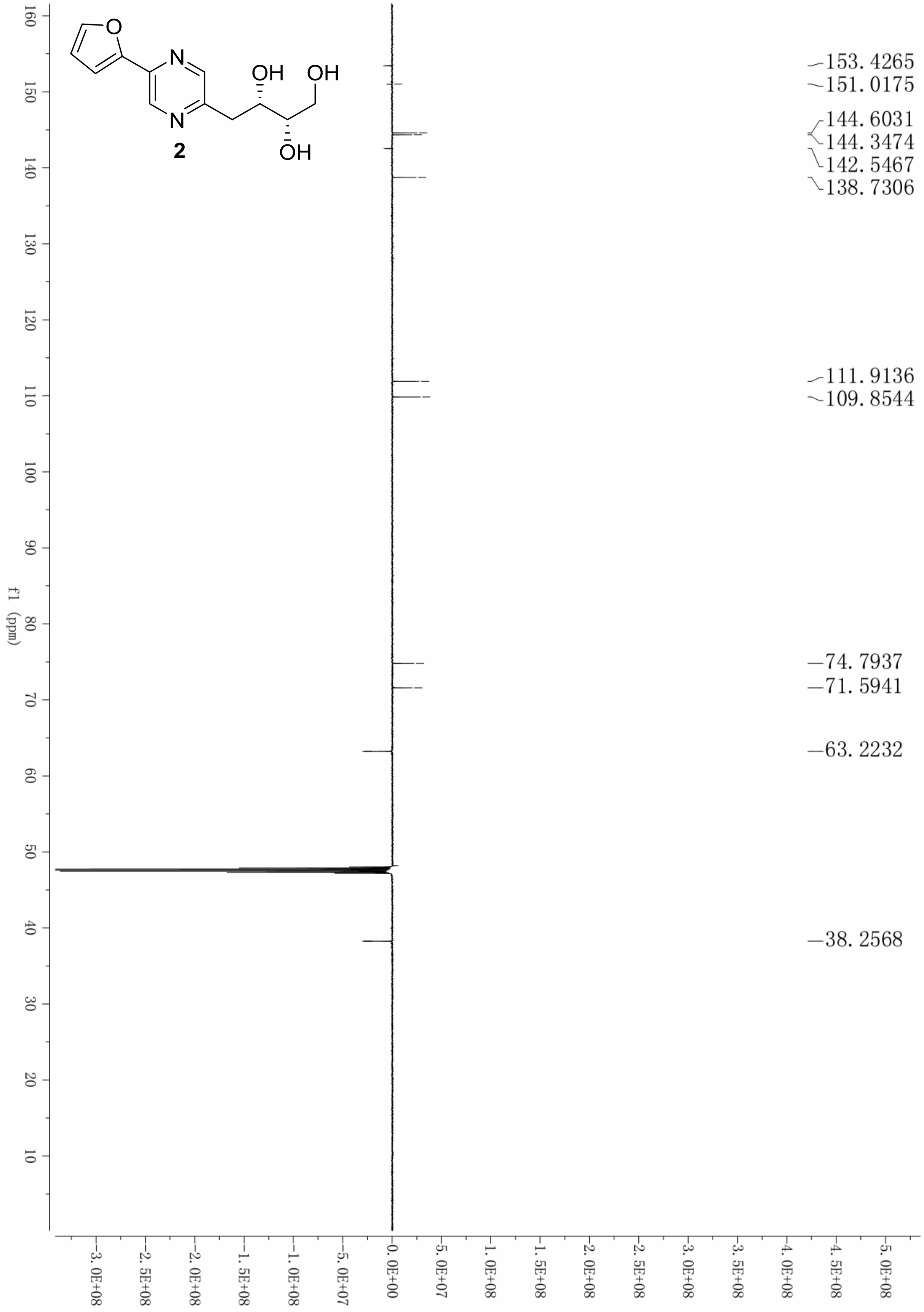

Figure S12. The ^{1}H-^{1}H COSY(600 MHz, MeOH-d_4) spectrum of compound **2**.

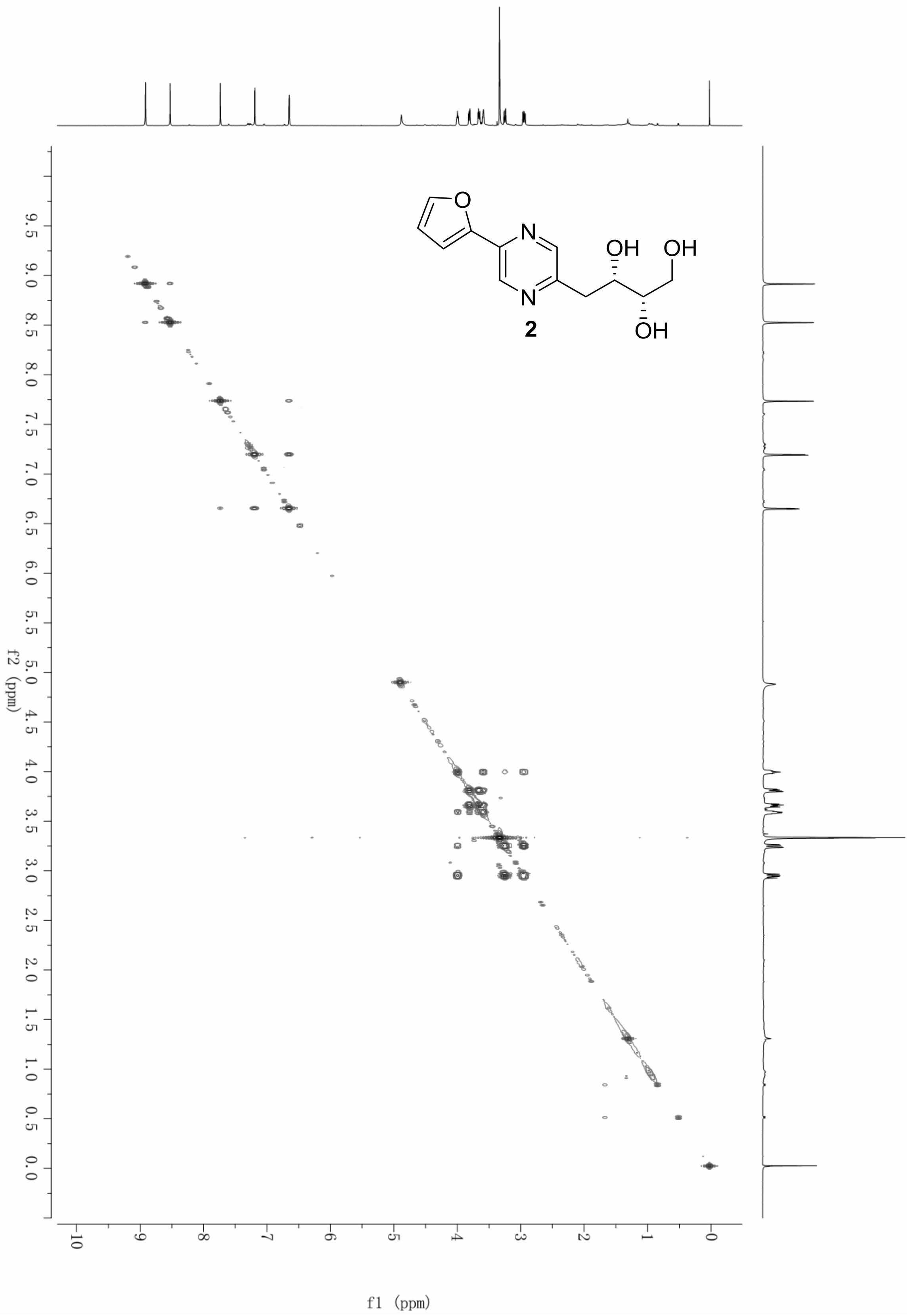

Figure S13. The HSQC (150 MHz, MeOH-d_4) spectrum of compound **2**.

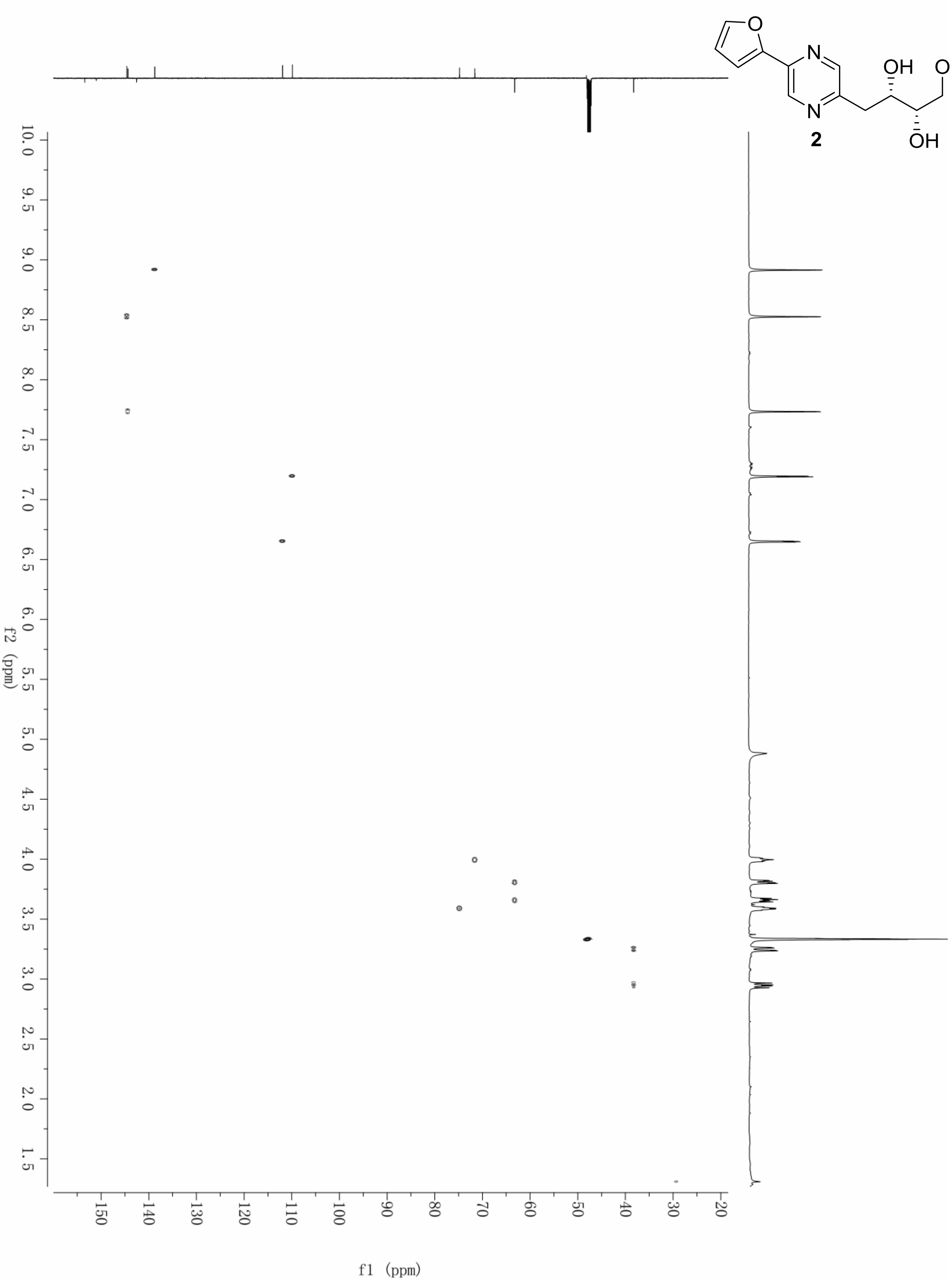

Figure S14. The HMBC (150 MHz, MeOH-d_4) spectrum of compound **2**.

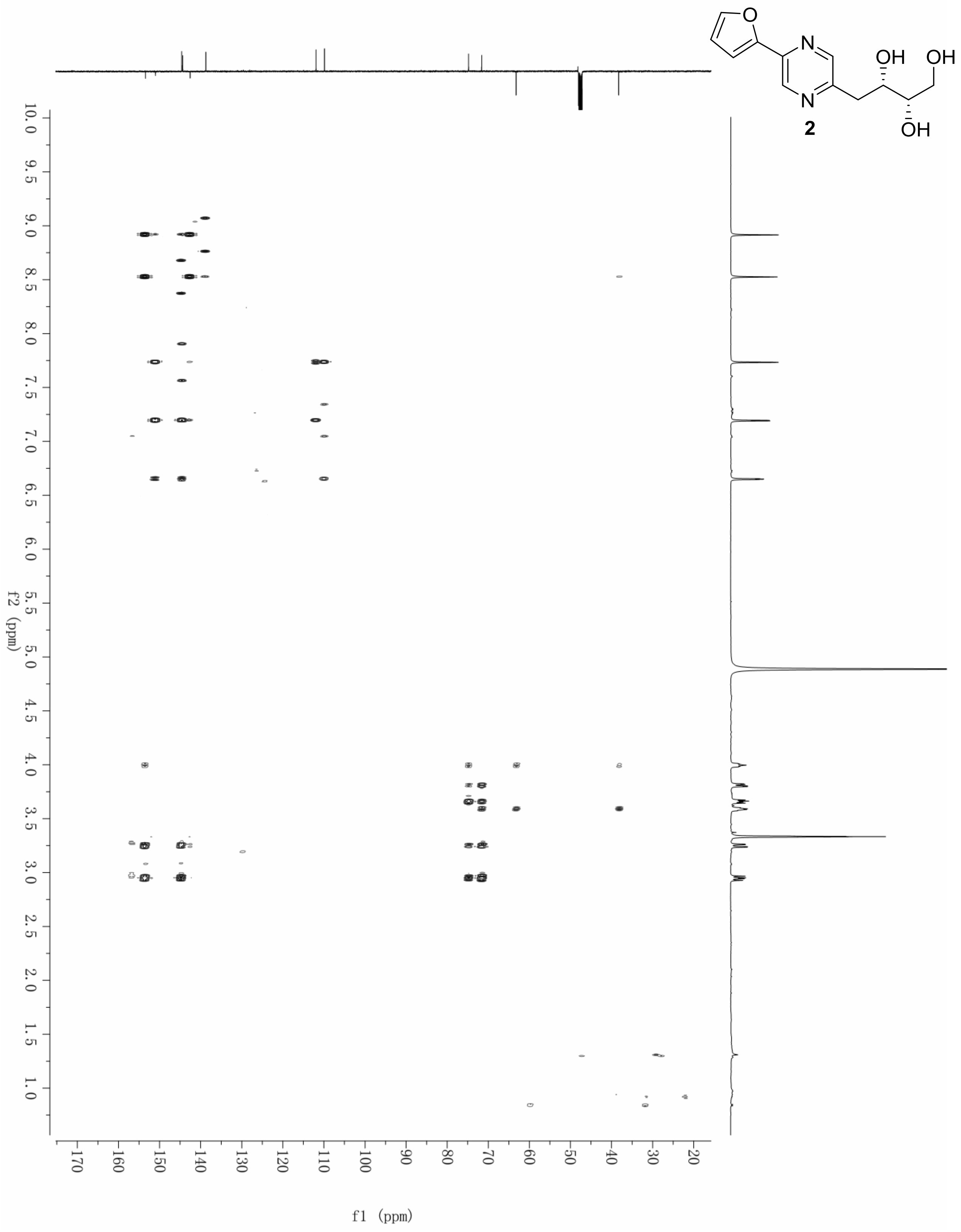

Figure S15. The NOESY (600 MHz, MeOH-d_4) spectrum of compound **2**.

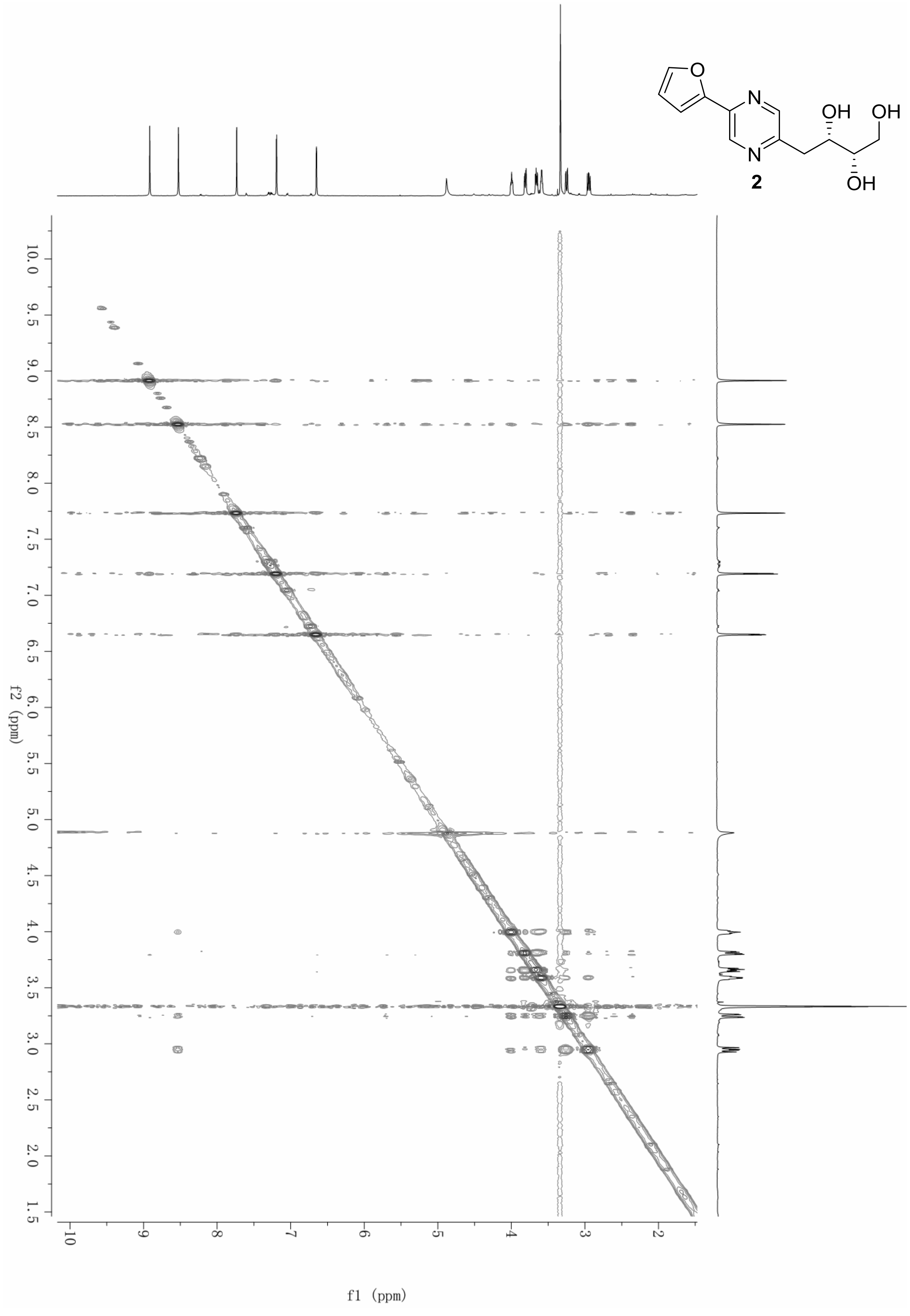

Figure S16. The NOESY (600 MHz, MeOH-d_4) spectrum of compound **2** at −4 ℃.

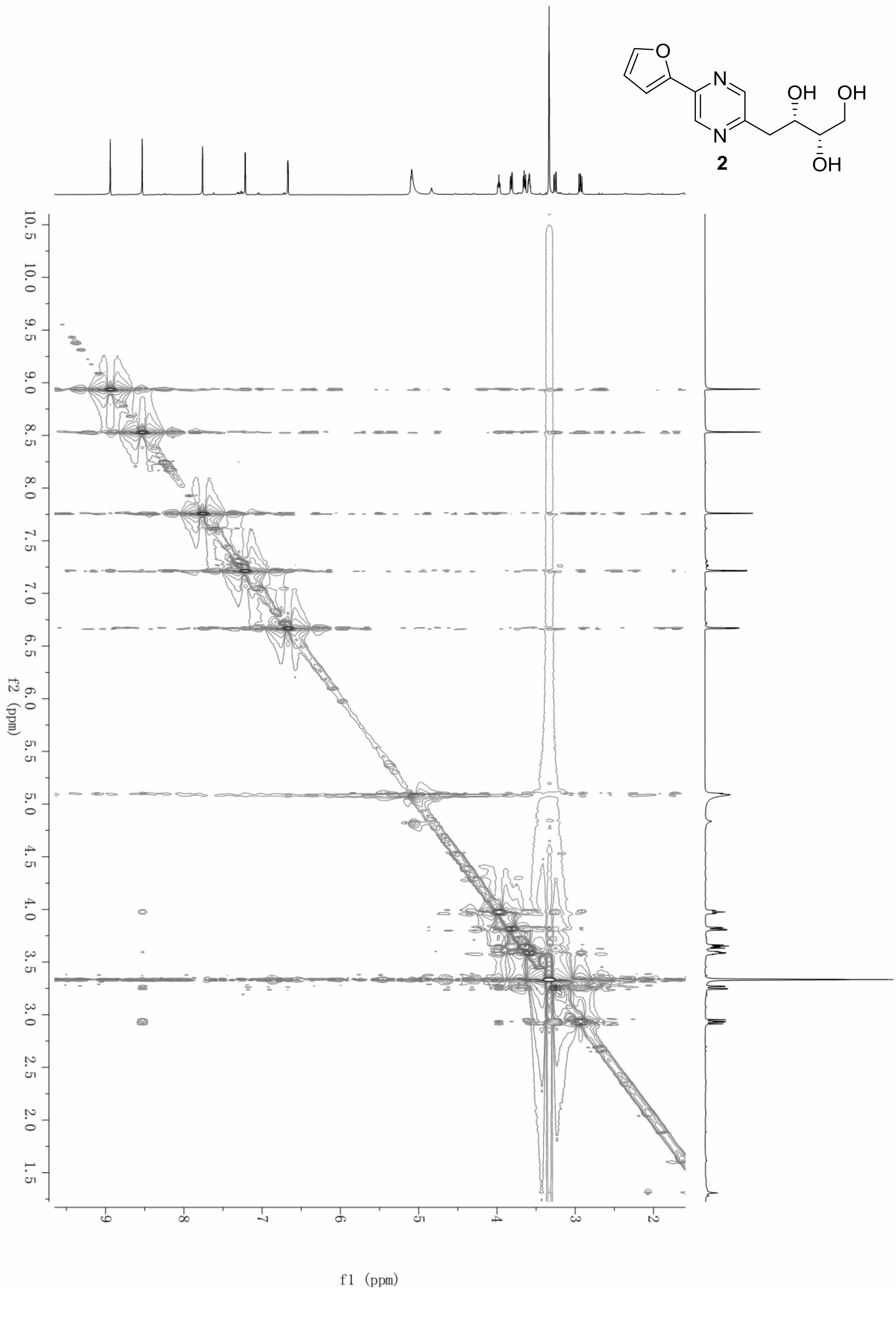

Figure S17. The ^{1}H NMR (600 MHz, DMSO-d_6) spectrum of compound **3**.

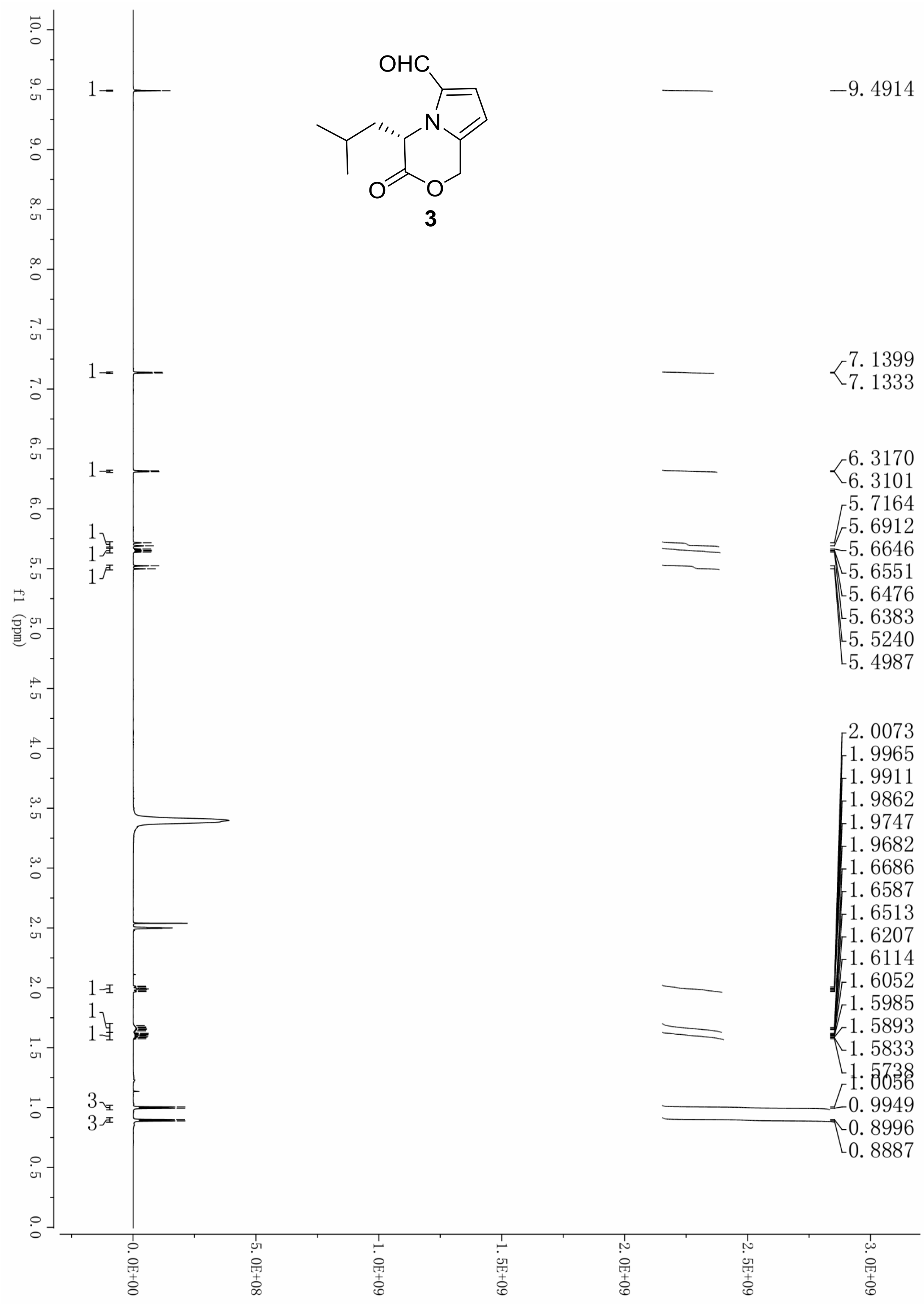

Figure S18. The ^{13}C NMR (150 MHz, DMSO-d_6) spectrum of compound **3**.

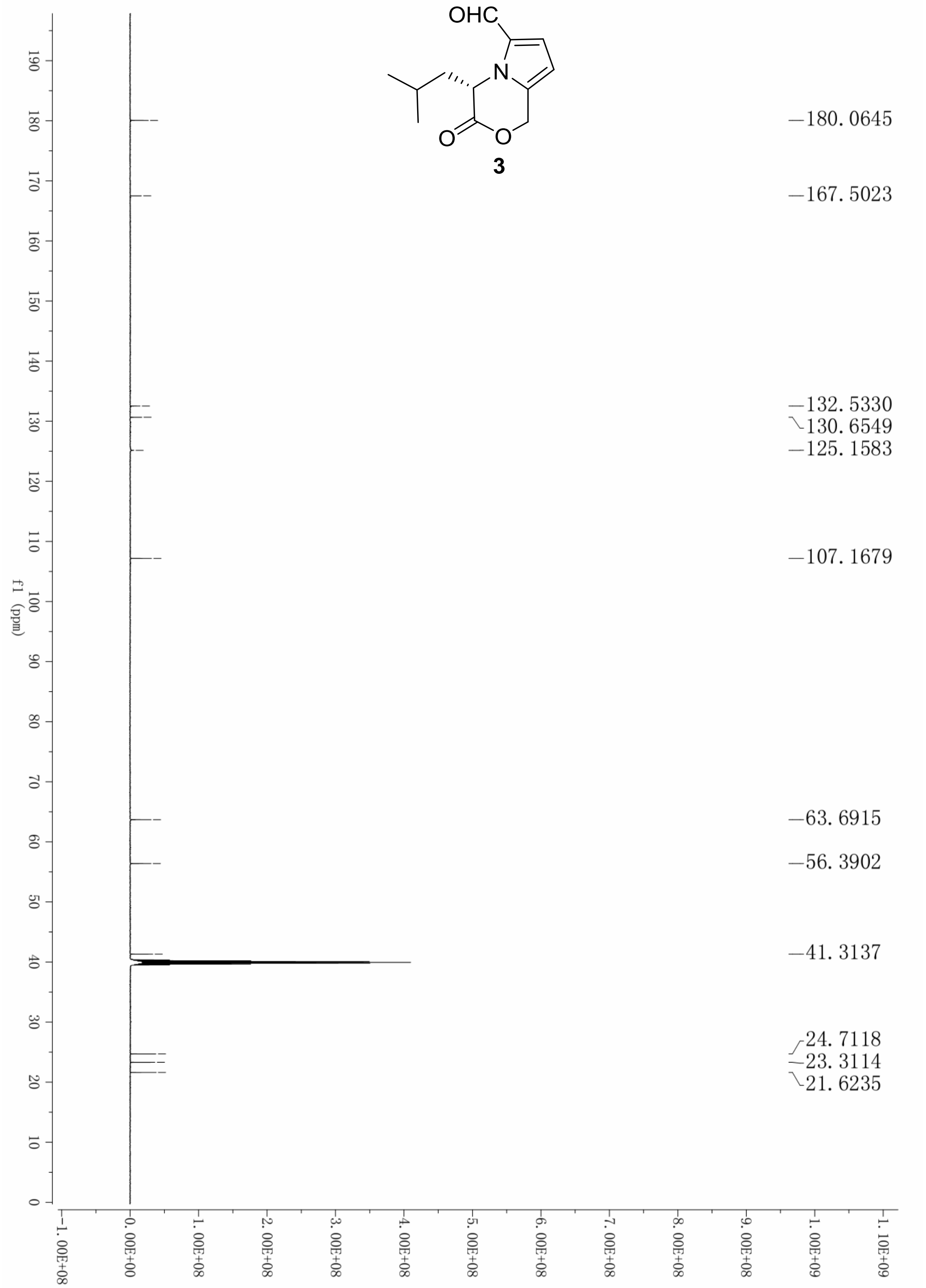

Figure S19. The DEPT (150 MHz, DMSO-d_6) spectrum of compound **3**.

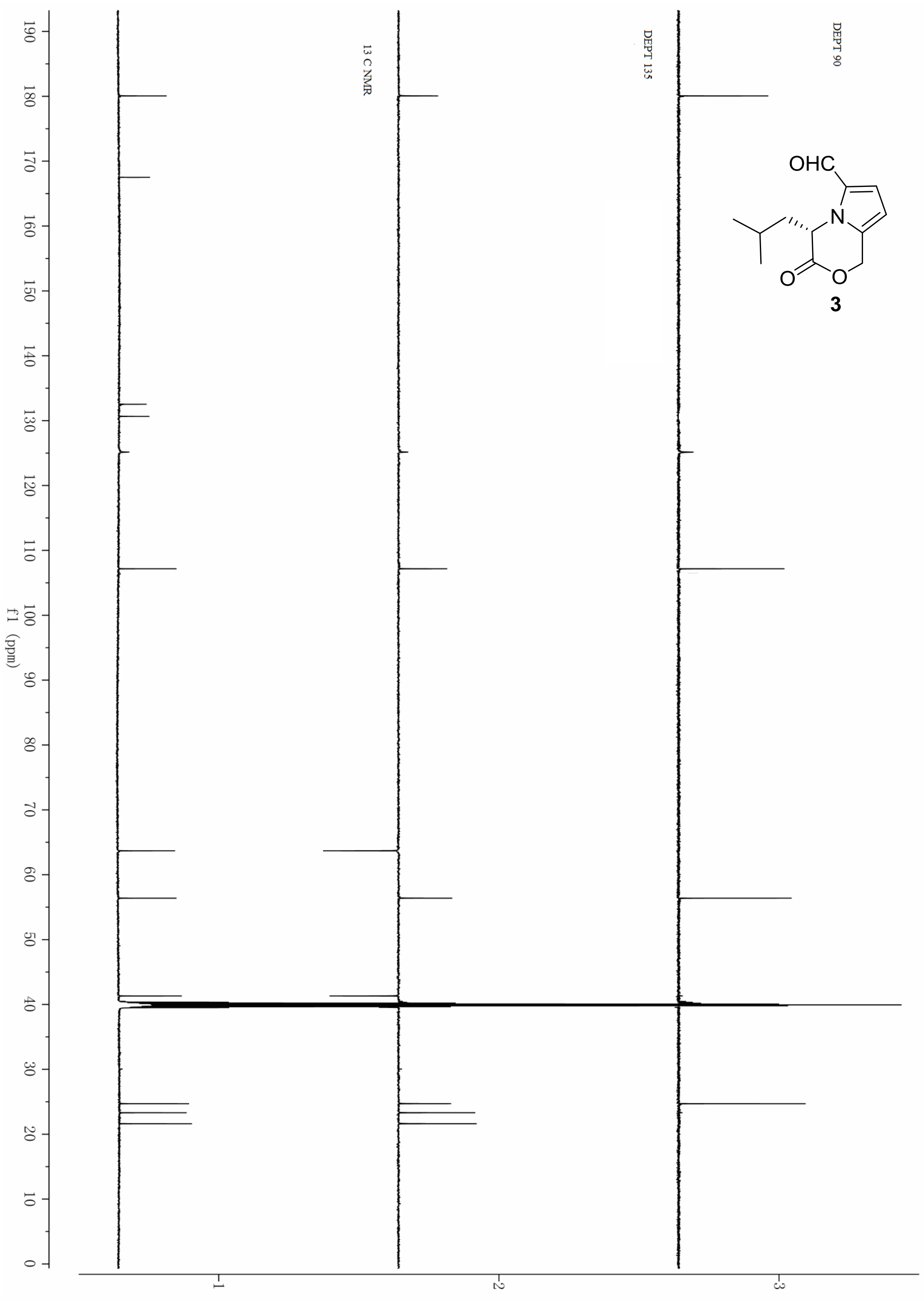

Figure S20. The ^{1}H-^{1}H COSY (600 MHz, DMSO-d_6) spectrum of compound **3**.

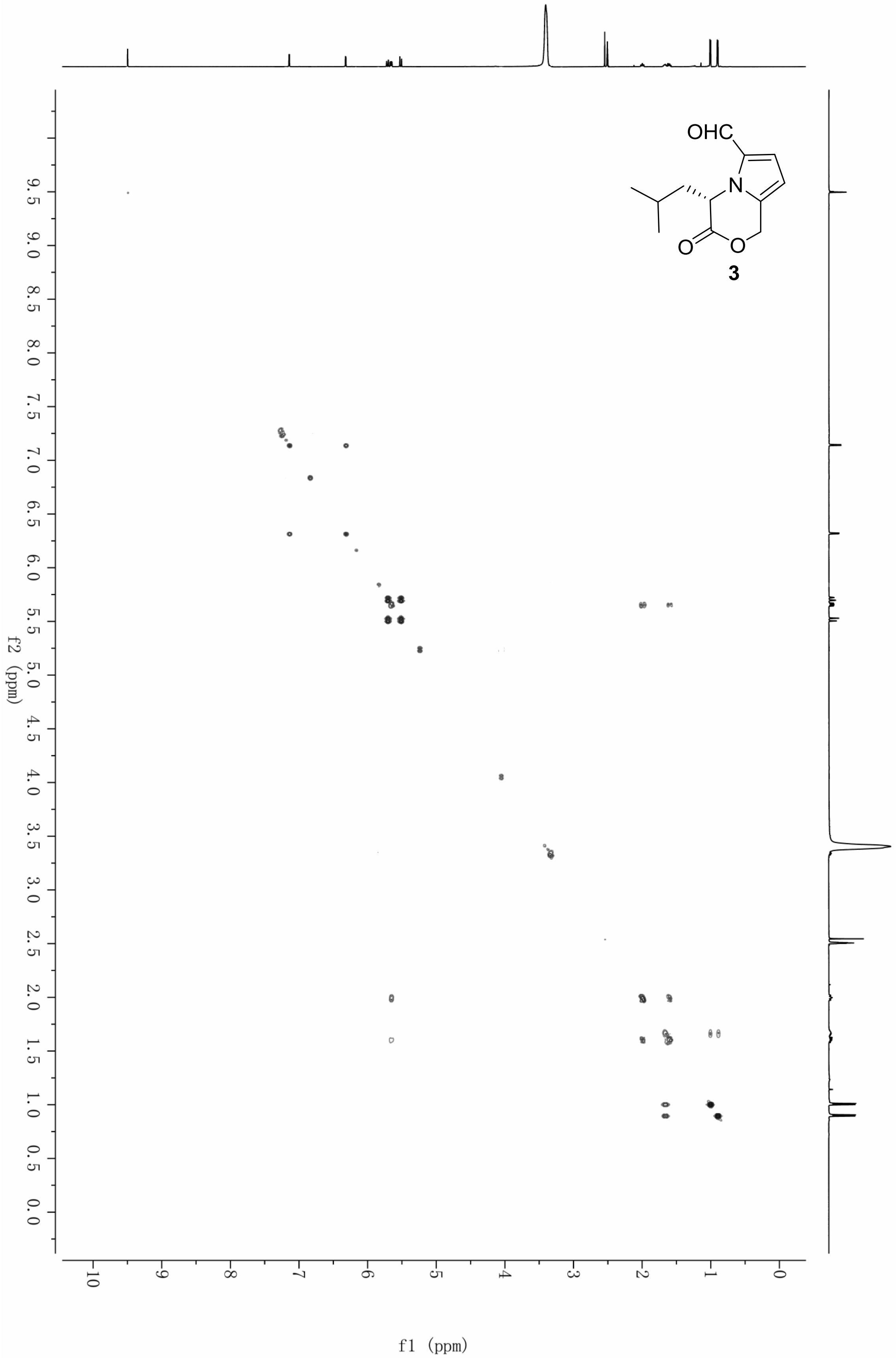

Figure S21. The HSQC (150 MHz, DMSO-d_6) spectrum of compound **3**.

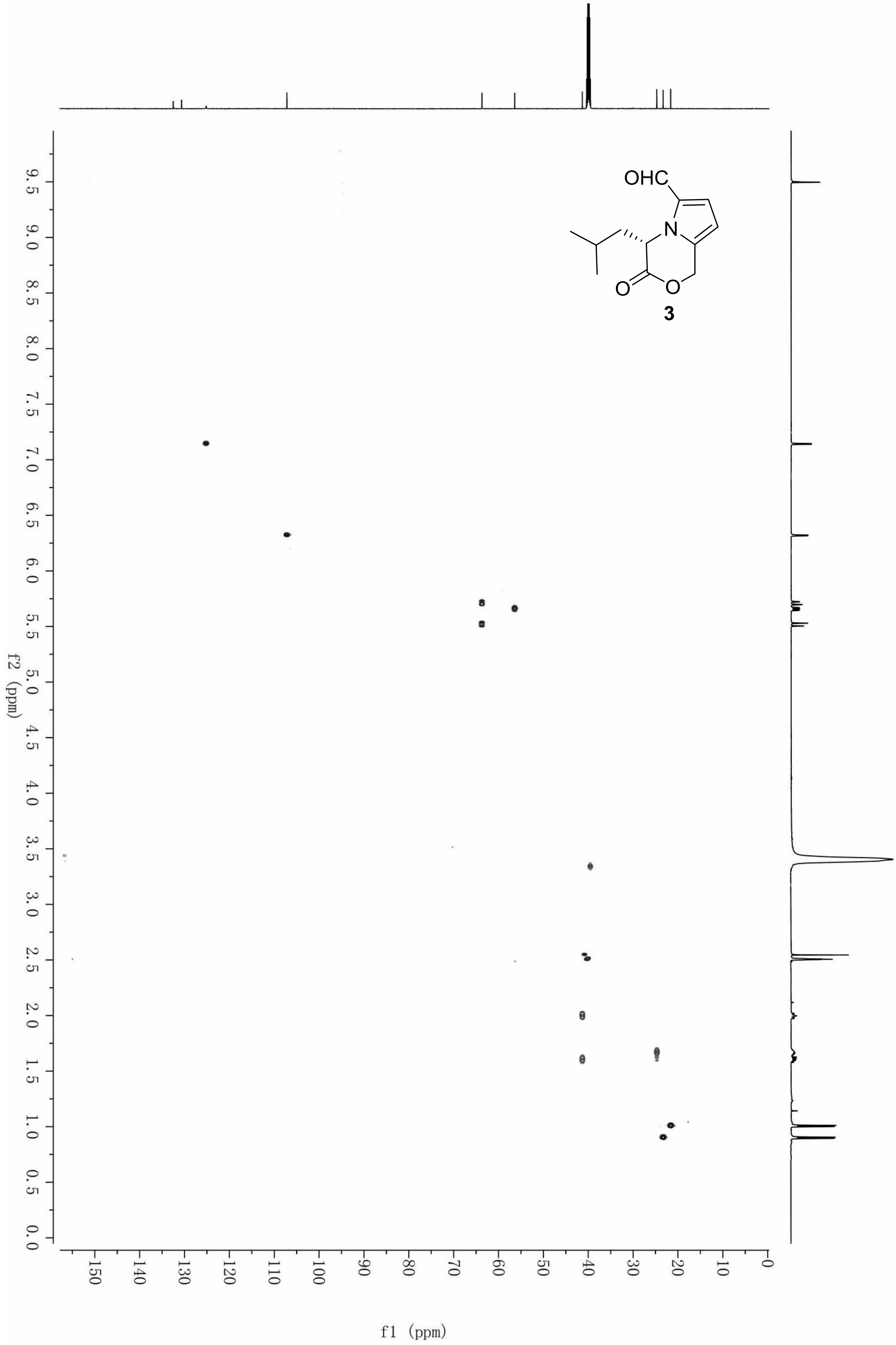

Figure S22. The HMBC (150 MHz, DMSO-d_6) spectrum of compound **3**.

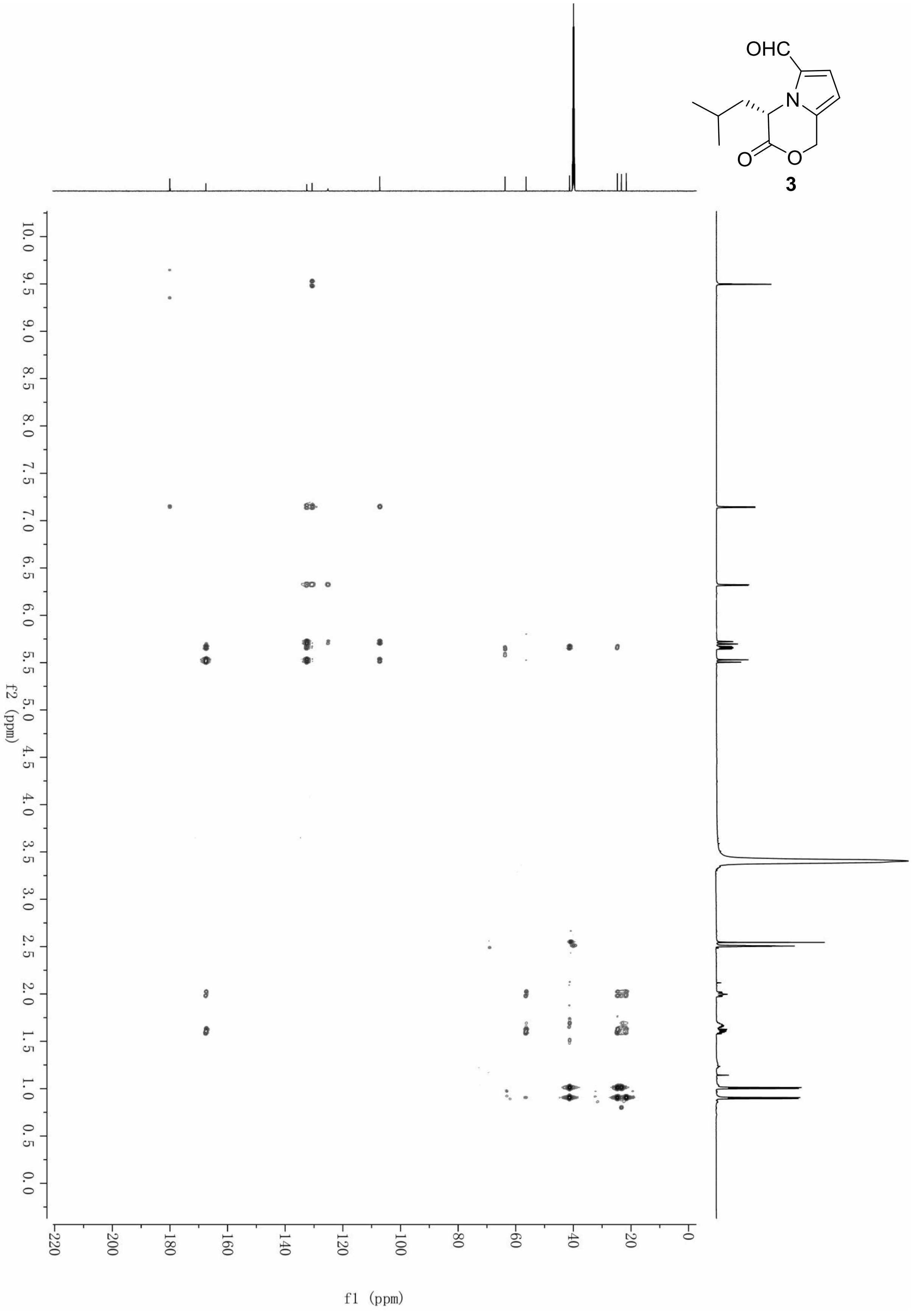

Figure S23. The ^{1}H NMR (600 MHz, DMSO-d_6) spectrum of compound **4**.

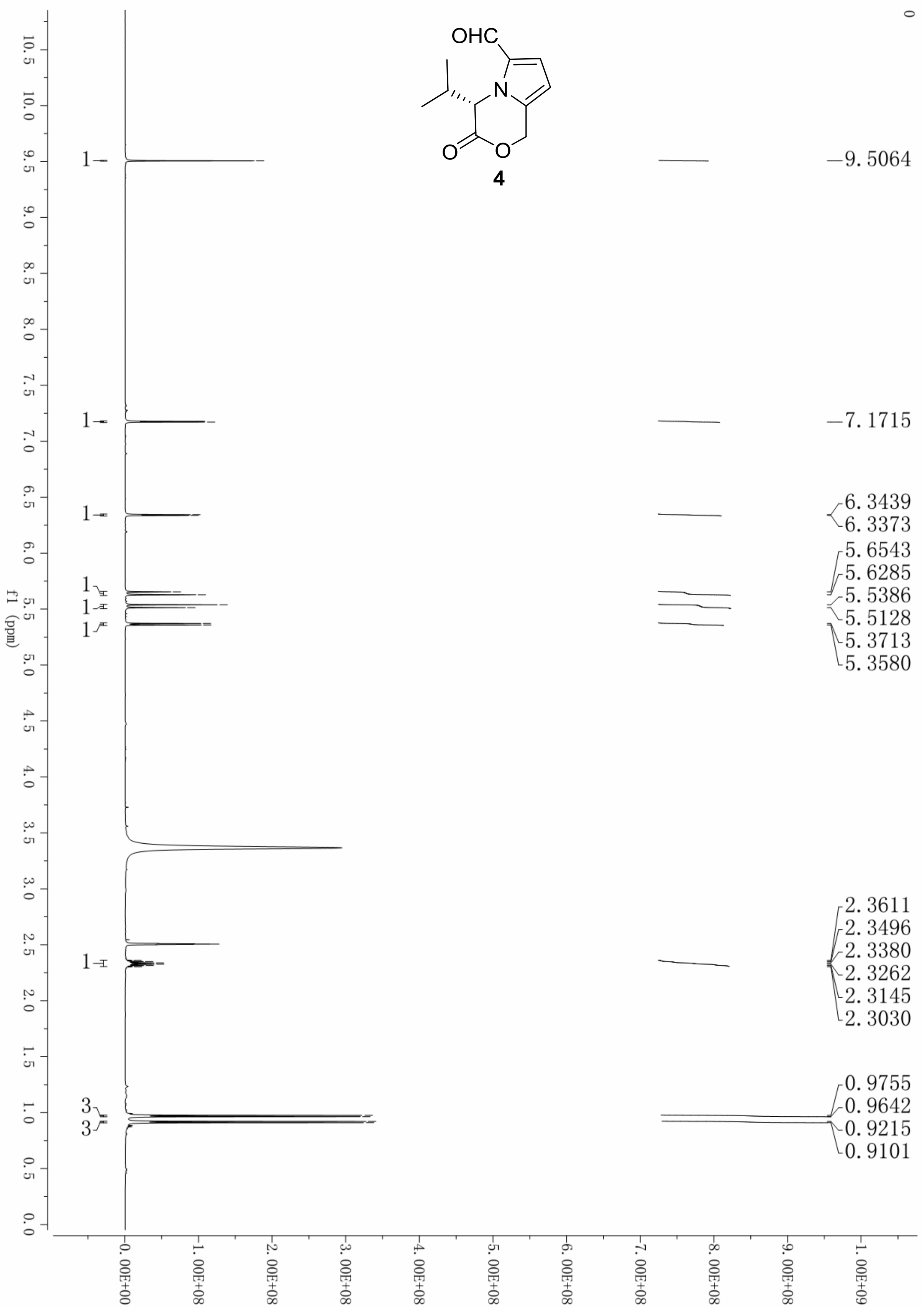

Figure S24. The ^{13}C NMR (150 MHz, DMSO-d_6) spectrum of compound **4**.

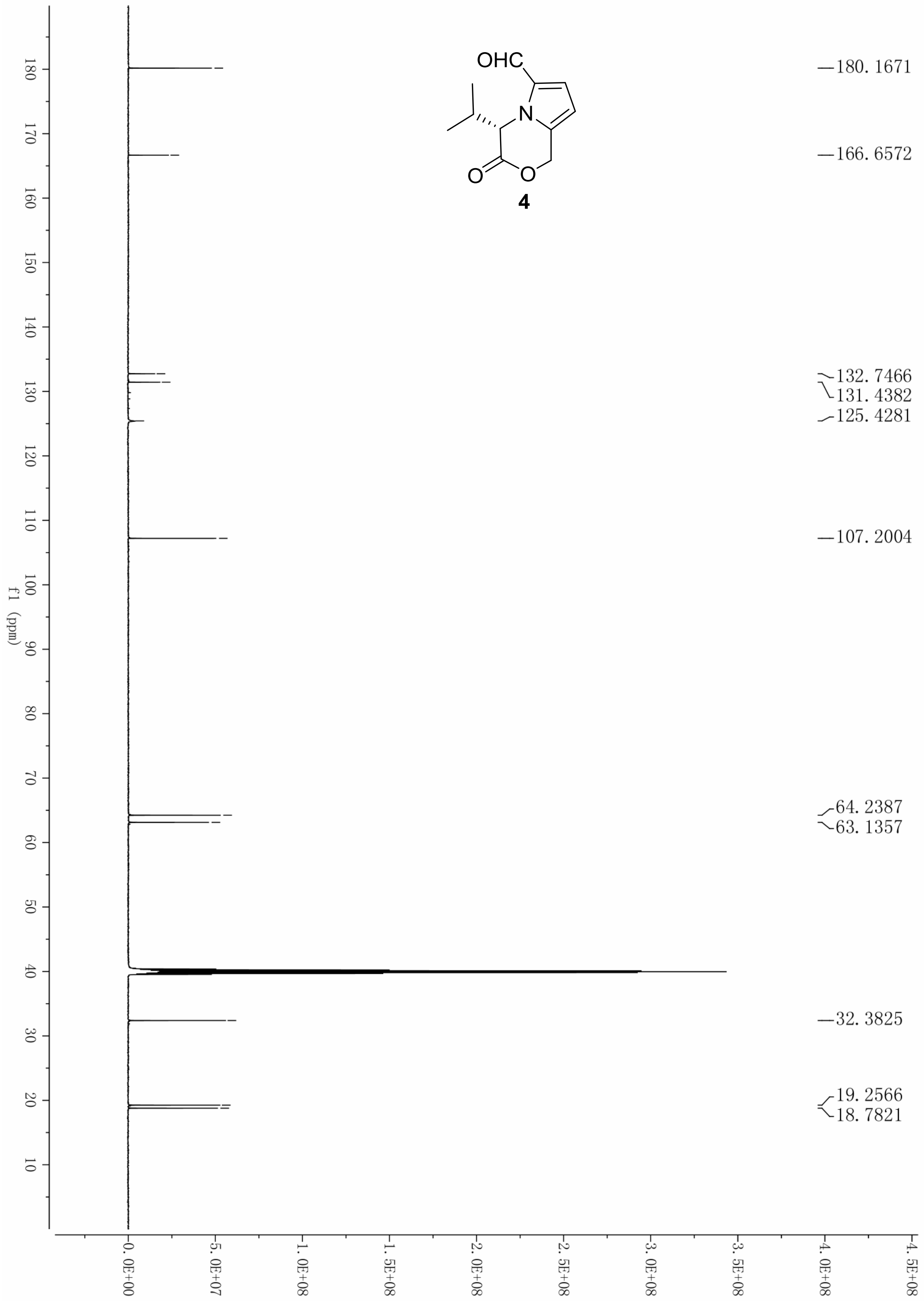

Figure S25. The DEPT (150 MHz, DMSO-d_6) spectrum of compound **4**.

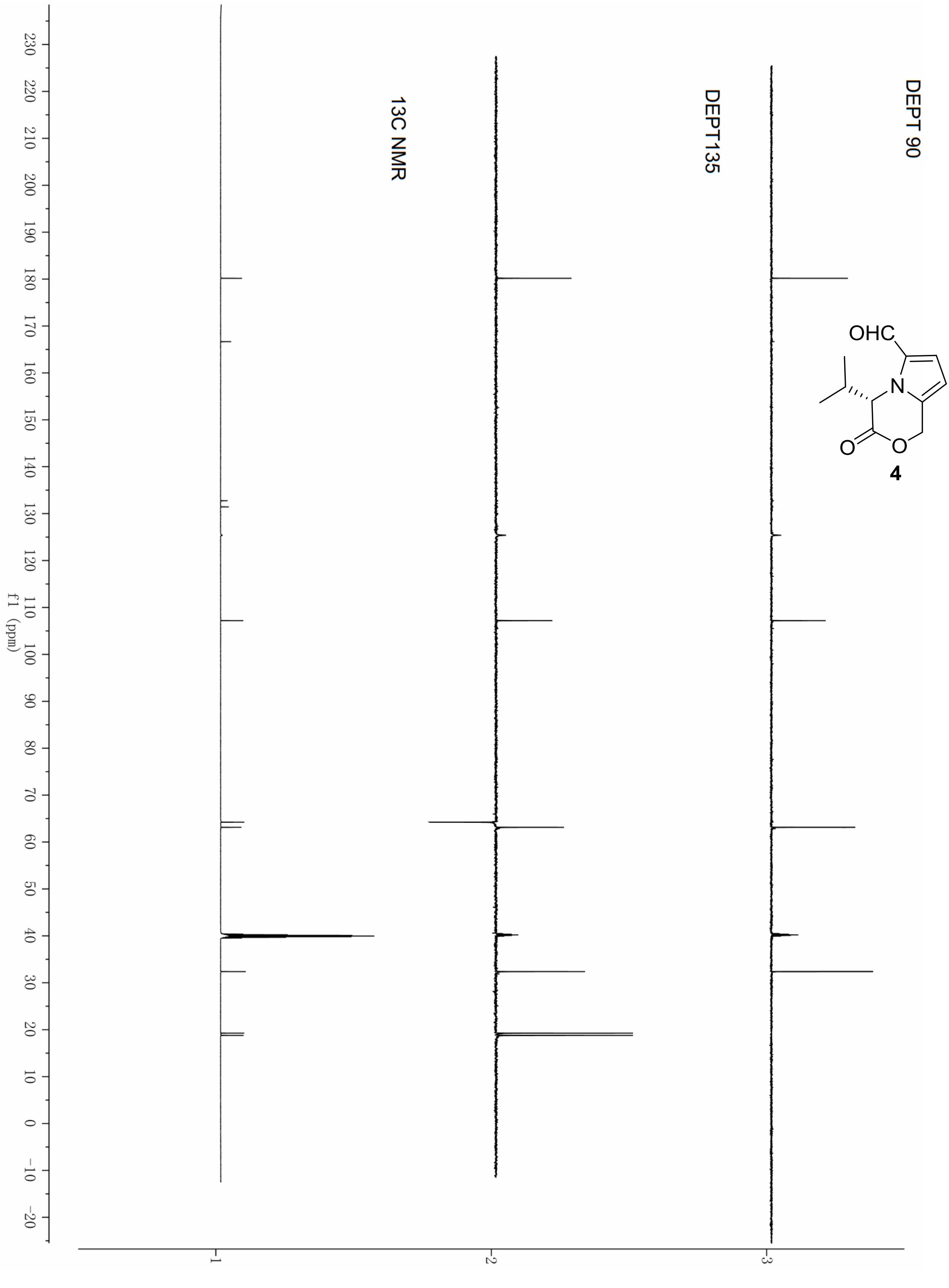

Figure S26. The ^{1}H NMR (600 MHz, DMSO-d_6) spectrum of compound **5**.

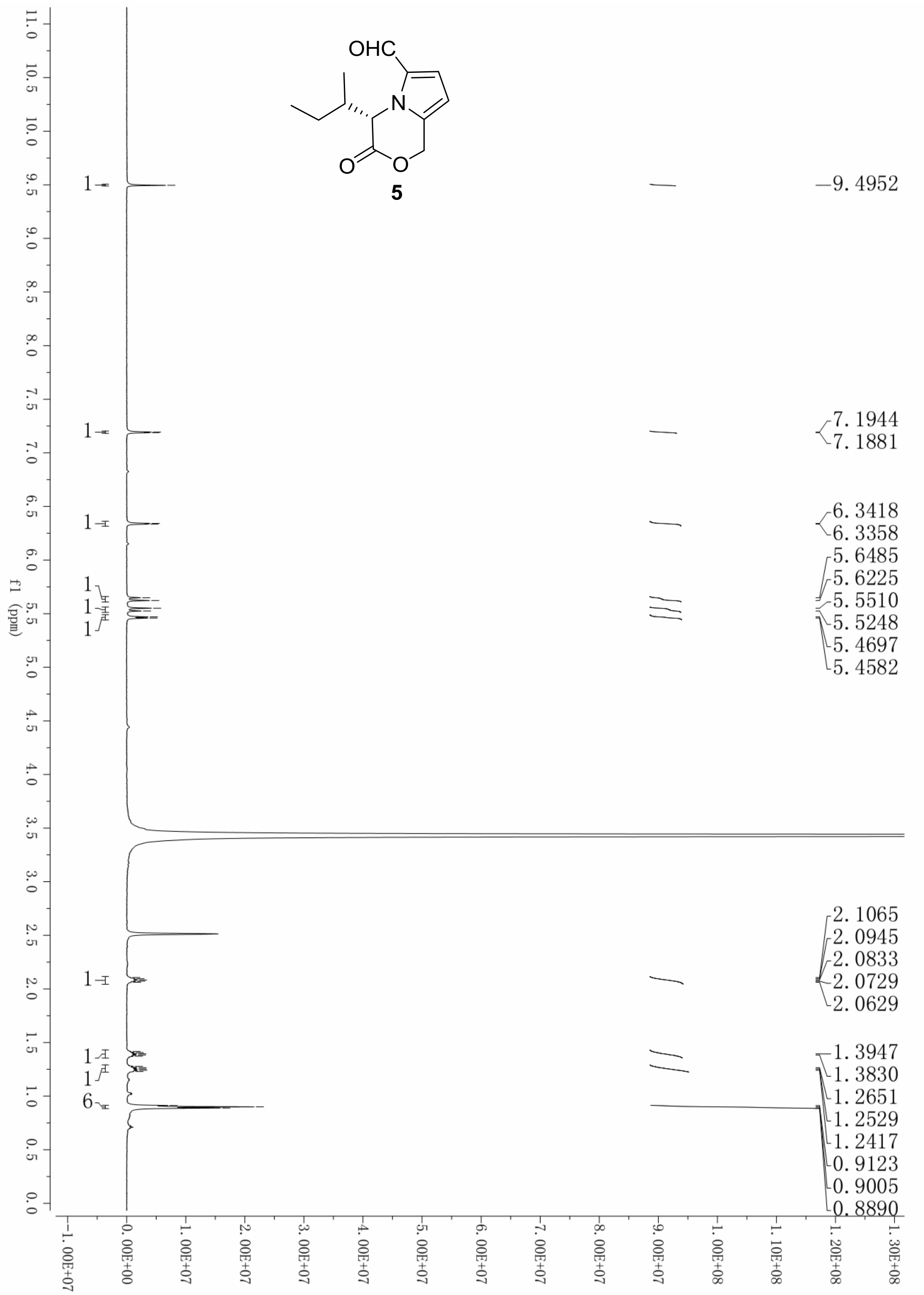

Figure S27. The DEPTQ (150 MHz, DMSO-d_6) spectrum of compound **5**.

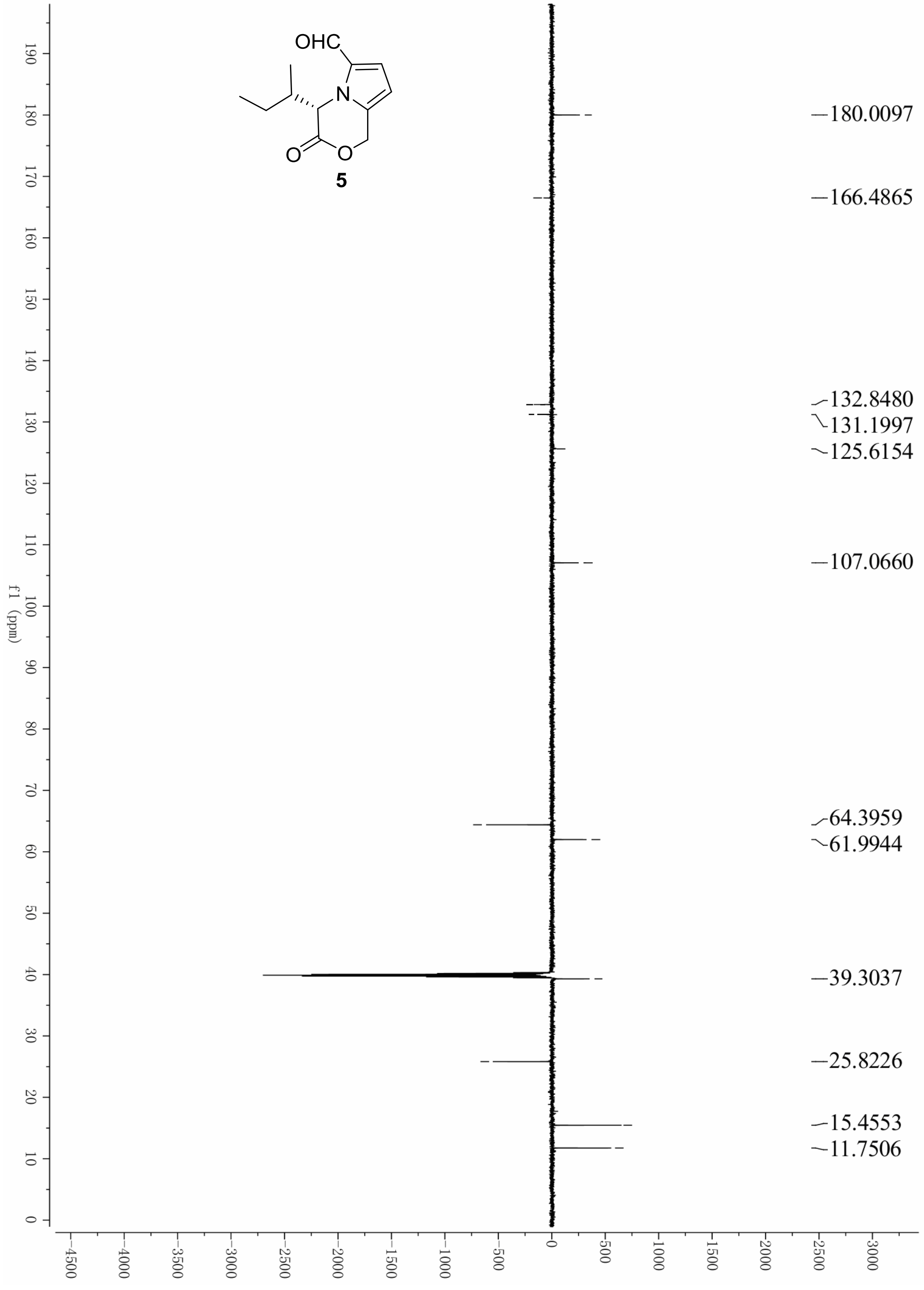

Figure S28. The ^{1}H NMR (600 MHz, DMSO-d_6) spectrum of compound **6**.

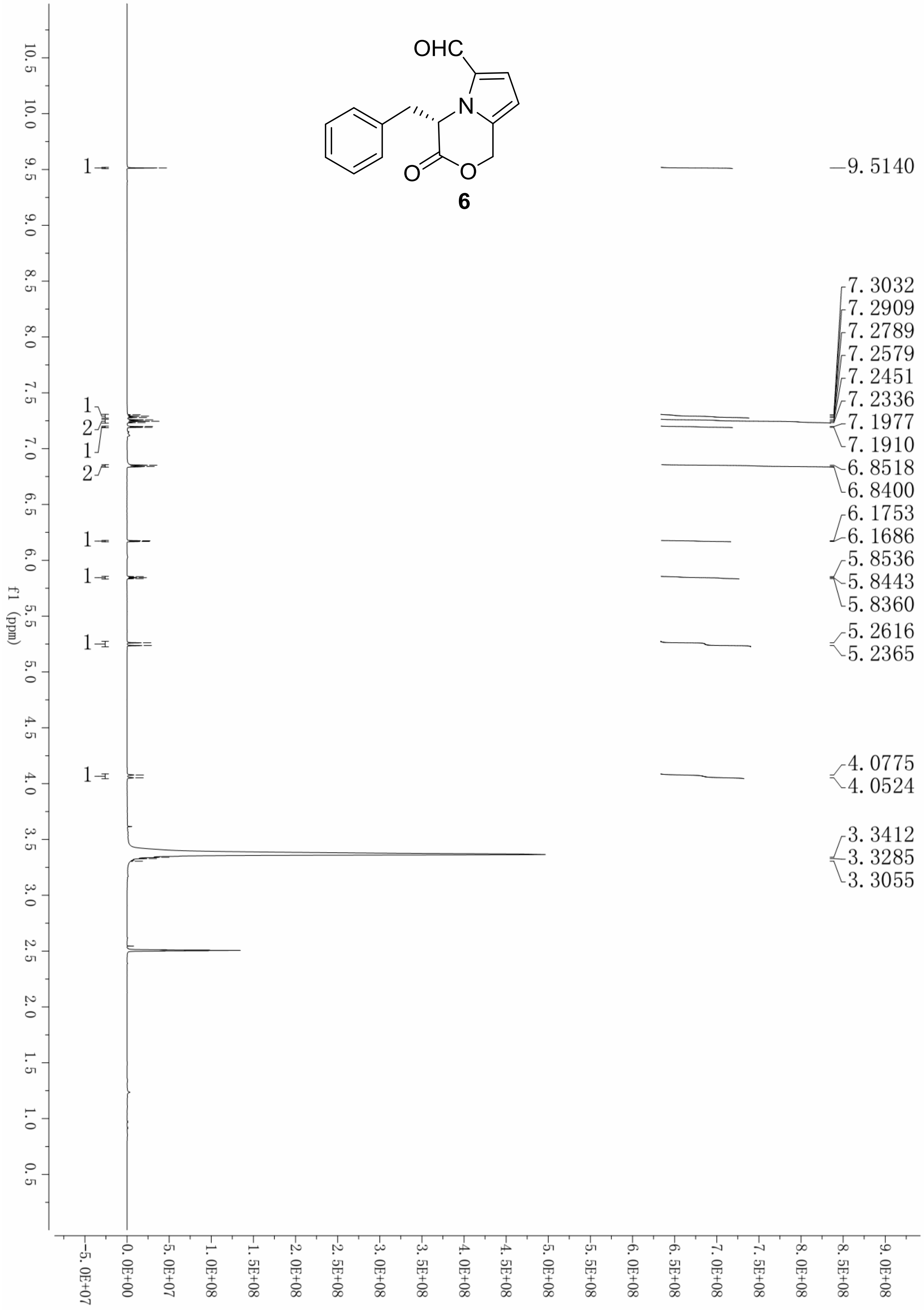

Figure S29. The ^{13}C NMR (150 MHz, DMSO-d_6) spectrum of compound **6**.

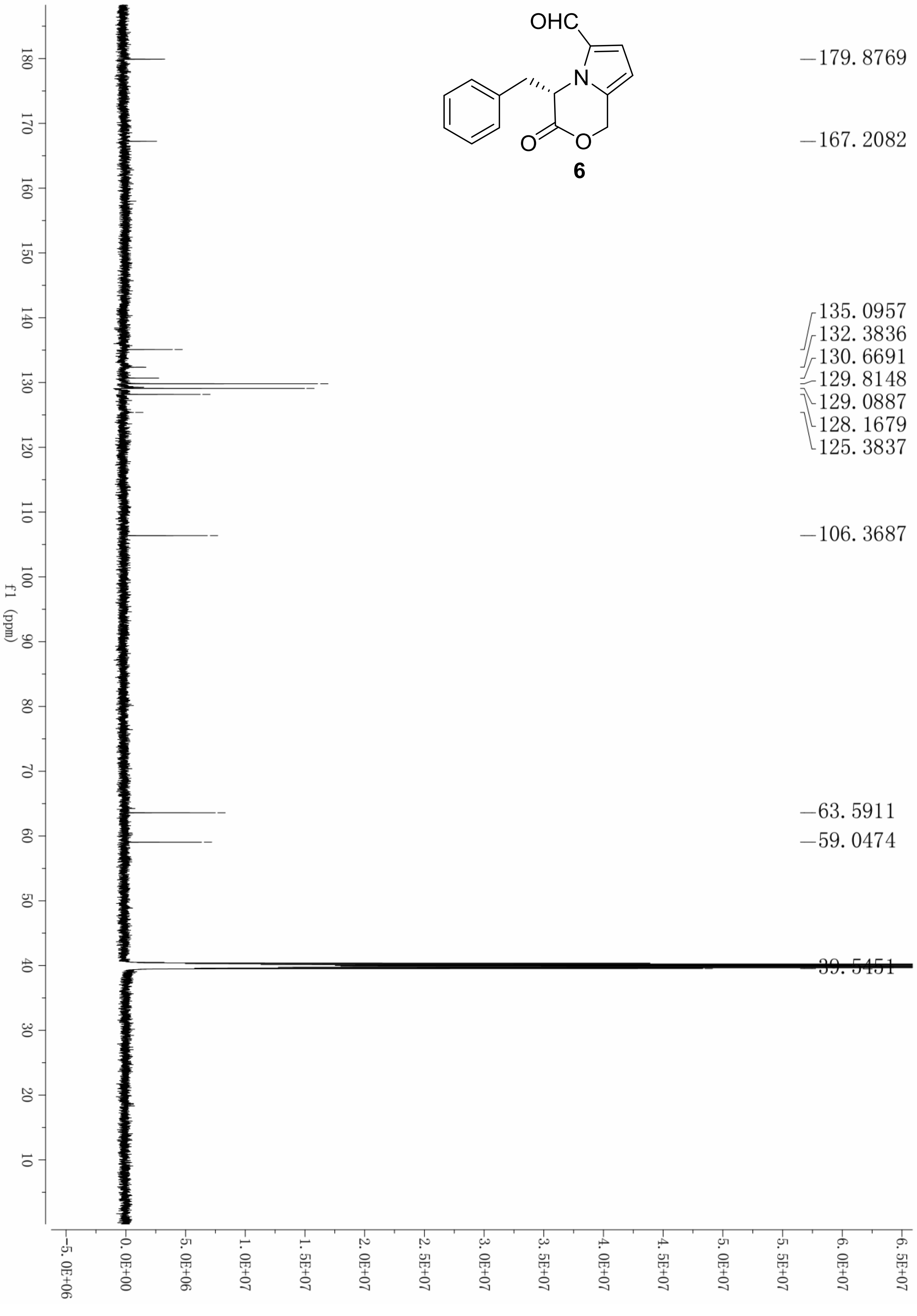

Figure S30. The DEPT (150 MHz, DMSO-d_6) spectrum of compound **6**.

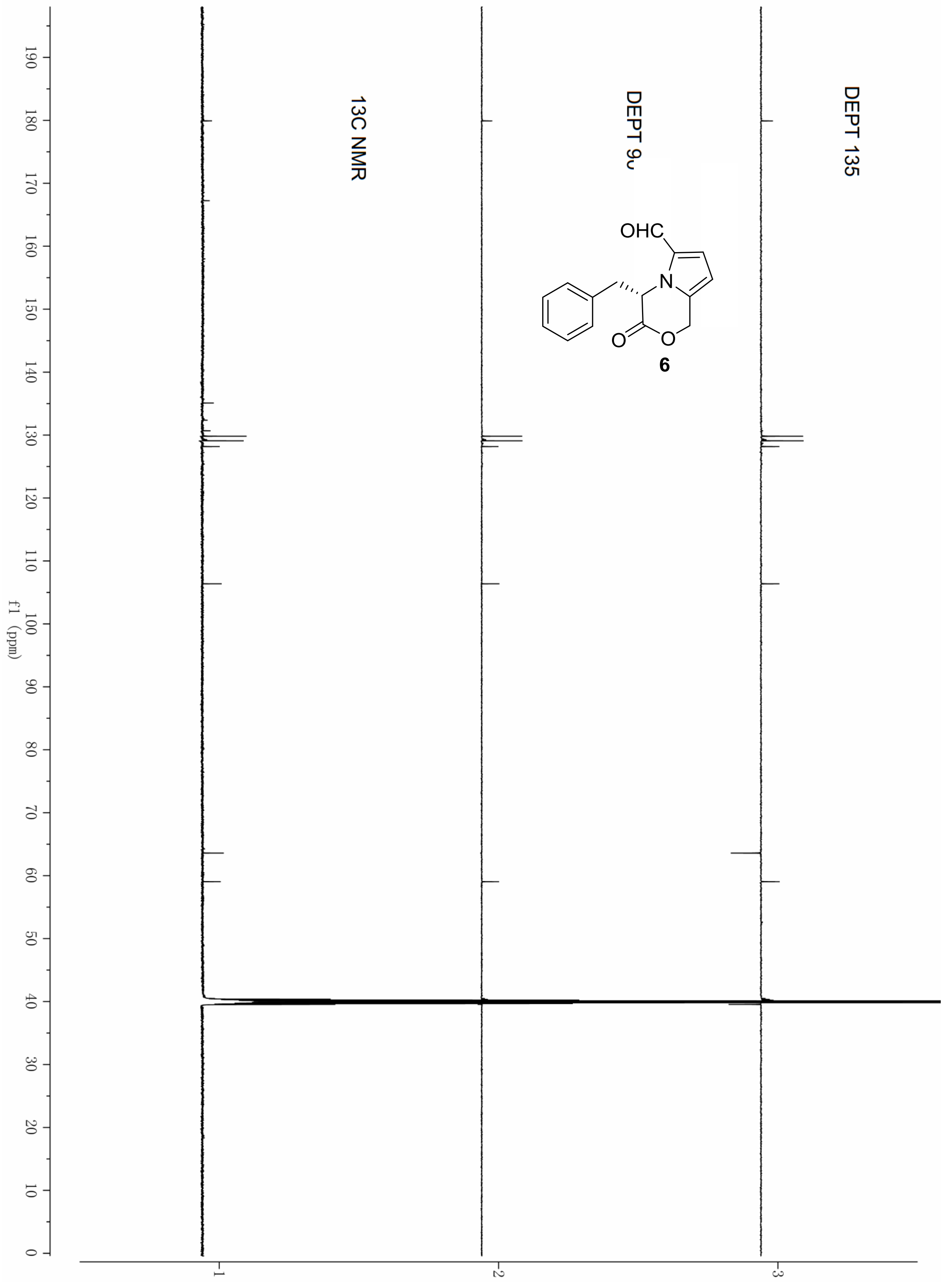

Table S1. Anti-H1N1 virus activities of **1**–**13**.

**Compounds**	**MDCK**	**H1N1**	
	**CC_{50} (μg/mL)**	**IC_{50}(μg/mL)**	**SI**
**1**	ND [a]	>50	ND [a]
**2**	ND [a]	>50	ND [a]
**3**	ND [a]	>50	ND [a]
**4**	ND [a]	>50	ND [a]
**5**	ND [a]	>50	ND [a]
**6**	ND [a]	>50	ND [a]
**7**	ND [a]	>50	ND [a]
**8**	116.3 ± 12.1	38.3 ± 1.2	3.0
**9**	403.2 ± 31.4	25.0 ± 3.6	16.1
**10**	124.1 ± 10.5	39.7 ± 5.6	3.1
**11**	522.5 ± 24.5	45.9 ± 2.1	11.4
**12**	ND [a]	>50	ND [a]
**13**	ND [a]	>50	ND [a]
Ribavirin	744.2 ± 18.5	23.1 ± 1.7	32.2

[a] Not detected.

Table S2. The 2D NMR data for compounds **1**–**3**.

**Position**	**1**			**2**			**Position**	**3**	
	**^1H-^1H COSY**	**HMBC**	**NOESY**	**^1H-^1H COSY**	**HMBC**	**NOEY**		**^1H-^1H COSY**	**HMBC**
1							1		
2							2		
3		2′, 2, 5		6	2, 5		3		2, 8, 11,12
4							4		
5		1″, 3, 6	1″, 2″				5		
6				3	2, 5, 1″	1″, 2″	6	7	5, 7, 8 , 10
1′							7		5, 6, 8, 9
2′							8		
3′	4′	2′, 4′, 5′		4′	2, 2′, 4′, 5		9		2, 6, 7, 8
4′	3′, 5′	2′, 3′, 5′		3′, 5′	2′, 3′, 5′		10		5
5′	4′	2′, 3′		4′	2′, 3′, 4′		11	3, 12,	2, 3, 12, 13
1″	2″	5, 6, 2″, 3″	3″, 4″	2″	5, 6, 2″, 3″	3″, 4″	12	11, 13, 14	3, 11, 13, 14
2″	1″, 3″	1″, 3″, 4″, 5, 6	5, 4″	1″, 3″	5, 1″, 3″, 4″	5, 4″	13	12	11, 12, 14
3″	2″, 4″	1″, 2″, 4″	1″	1″, 4″	1″, 2″, 4″	1″	14	12	11, 12, 13
4″	3″	2″, 3″	1″, 2″	3″	2″, 3″	1″			

© 2014 by the authors; licensee MDPI, Basel, Switzerland. This article is an open access article distributed under the terms and conditions of the Creative Commons Attribution license (http://creativecommons.org/licenses/by/3.0/).
